# Molecular structure, DNA binding mode, photophysical properties and recommendations for use of SYBR Gold

**DOI:** 10.1093/nar/gkab265

**Published:** 2021-04-27

**Authors:** Pauline J Kolbeck, Willem Vanderlinden, Gerd Gemmecker, Christian Gebhardt, Martin Lehmann, Aidin Lak, Thomas Nicolaus, Thorben Cordes, Jan Lipfert

**Affiliations:** Department of Physics and Center for NanoScience, LMU Munich, Amalienstrasse 54, 80799 Munich, Germany; Department of Physics and Center for NanoScience, LMU Munich, Amalienstrasse 54, 80799 Munich, Germany; Bavarian NMR Center (BNMRZ), Department of Chemistry, Technical University of Munich, Garching, Germany; Physical and Synthetic Biology, Faculty of Biology, LMU Munich, Planegg-Martinsried, Germany; Plant Molecular Biology, Faculty of Biology, LMU Munich, Planegg-Martinsried, Germany; Department of Physics and Center for NanoScience, LMU Munich, Amalienstrasse 54, 80799 Munich, Germany; Department of Physics and Center for NanoScience, LMU Munich, Amalienstrasse 54, 80799 Munich, Germany; Physical and Synthetic Biology, Faculty of Biology, LMU Munich, Planegg-Martinsried, Germany; Department of Physics and Center for NanoScience, LMU Munich, Amalienstrasse 54, 80799 Munich, Germany

## Abstract

SYBR Gold is a commonly used and particularly bright fluorescent DNA stain, however, its chemical structure is unknown and its binding mode to DNA remains controversial. Here, we solve the structure of SYBR Gold by NMR and mass spectrometry to be [2-(4-{[diethyl(methyl)ammonio]methyl}phenyl)-6-methoxy-1-methyl-4-{[(2Z)-3-methyl-1,3-benzoxazol-2-ylidene]methyl}quinolin-1-ium] and determine its extinction coefficient. We quantitate SYBR Gold binding to DNA using two complementary approaches. First, we use single-molecule magnetic tweezers (MT) to determine the effects of SYBR Gold binding on DNA length and twist. The MT assay reveals systematic lengthening and unwinding of DNA by 19.1° ± 0.7° per molecule upon binding, consistent with intercalation, similar to the related dye SYBR Green I. We complement the MT data with spectroscopic characterization of SYBR Gold. The data are well described by a global binding model for dye concentrations ≤2.5 μM, with parameters that quantitatively agree with the MT results. The fluorescence increases linearly with the number of intercalated SYBR Gold molecules up to dye concentrations of ∼2.5 μM, where quenching and inner filter effects become relevant. In summary, we provide a mechanistic understanding of DNA-SYBR Gold interactions and present practical guidelines for optimal DNA detection and quantitative DNA sensing applications using SYBR Gold.

## INTRODUCTION

The interaction of DNA with ligands is fundamental for many cellular processes as well as biotechnological applications. In particular, fluorescent dyes are routinely used to label DNA for visualization and quantification in a wide variety of assays ranging from imaging of cells to analysis and quantification of gel bands or PCR products. SYBR Gold is a popular stain with very high sensitivity owing to the >1000-fold increase in fluorescence quantum yield on binding to DNA (1,2). Despite its widespread use, its structure is unknown and there is disagreement whether SYBR Gold binds in an intercalative (2,3) or in a minor-groove binding mode (4–6).

In general, the binding mode of DNA dyes impacts how binding depends on environmental conditions, DNA chain topology, or sequence context. Conversely, small-molecule binding to DNA can alter its structure and mechanical properties. Specifically, intercalation lengthens and unwinds the DNA helix (7,8), and the changes upon intercalation into DNA have been investigated at the single-molecule level using optical tweezers (3,9–21), AFM force spectroscopy (22,23), and magnetic tweezers (MT) (24–27). In contrast, minor groove binding has only much smaller effects, if any, on DNA length and winding angle (24).

Detection of DNA, e.g. after separation by gel electrophoresis, requires labeling and staining for visualization and quantitation. The extent of DNA binding depends both on DNA and dye concentration, as with any equilibrium binding reaction. We need to quantitatively understand the binding properties of SYBR Gold to DNA in order to determine conditions for high signal-to-noise detection and to obtain a linear relationship between the amount of DNA and fluorescence intensity, which is desirable for quantitation.

Here, we first solved the molecular structure of SYBR Gold by NMR and mass spectrometry, which allows us to determine the charge, molecular weight and molecular extinction coefficient. To determine the binding mode of SYBR Gold to DNA and to investigate the fluorescence response under varying DNA and SYBR Gold concentrations, we combined single-molecule micromanipulation experiments with a range of fluorescence measurements. We present a binding model that describes both the single-molecule manipulation and bulk fluorescence data quantitatively. Binding parameters, i.e. the binding constant (dissociation constant) *K*_d_ and the binding site size $n$, were determined independently from both single-molecule manipulation and fluorescence experiments and were found to quantitatively agree. The close agreement between binding parameters determined from micromanipulation and fluorescence strongly suggests that intercalation is the sole binding mode of SYBR Gold that enhances fluorescence upon DNA binding. In addition, we performed single-molecule binding measurements for the closely related compound SYBR Green I, which we find to intercalate with binding parameters very similar to SYBR Gold.

For SYBR Gold, we observe a reduction of fluorescence intensity at SYBR Gold concentrations >2.5 μM (for typical experimental path lengths ∼5–10 mm) and distinguish quenching mechanisms by fluorescence lifetime measurements. Based on our experimental results, we present practical guidelines for optimal DNA detection and quantitative DNA sensing applications using SYBR Gold.

## MATERIALS AND METHODS

### Chemicals and concentration determination

SYBR Gold and SYBR Green I were purchased as ‘0.5 ml 10 000× concentrate in DMSO’ from Invitrogen. For SYBR Green I, we determined the absorbance at 495 nm of the stock solution to be 585 ± 35 (the error is from the uncertainty of the fit to data from a serial dilution) and used the published (28) molecular extinction coefficient ϵ_495 nm_ = 58 000 M^−1^cm^−1^ to determine the stock concentration to be 10.1 ± 0.6 mM. For SYBR Gold, no molecular extinction coefficient had been reported and the concentration of the stock solution is not specified by the vendor. We therefore determined the concentration and molecular extinction coefficient by lyophilizing the stock solution (2 days at 0.04 mbar) and weighing the dried sample. We determined the mass of the lyophilized stock to be 3.5 ± 0.4 mg (with a 10% uncertainty from sample handling). The mass of the SYBR Gold cation was determined to be 495.2 Da by mass spectrometry, see the next section. Assuming further two chloride ions as the relevant anions (29,30), we find a concentration of the stock solution of 12.4 ± 1.2 mM. From a serial dilution, we determined the absorbance of the lyophilized stock at 486 nm to be 703 ± 10 (Supplementary Figure S1), which gives a molar extinction coefficient for SYBR Gold of ϵ_486 nm_ = (57 000 ± 800) M^−1^ cm^−1^.

We found that the concentrations determined from absorbance measurements vary significantly (by ∼50%; greater than our measurement uncertainty) for different SYBR Gold stocks supplied by the same vendor (Supplementary Figure S1 and Supplementary Table S1). We, therefore, determined the concentrations for each stock solution used in the experiments separately from absorbance measurements in the absence of DNA (Supplementary Figure S1).

### Structure determination

**Figure 1.**
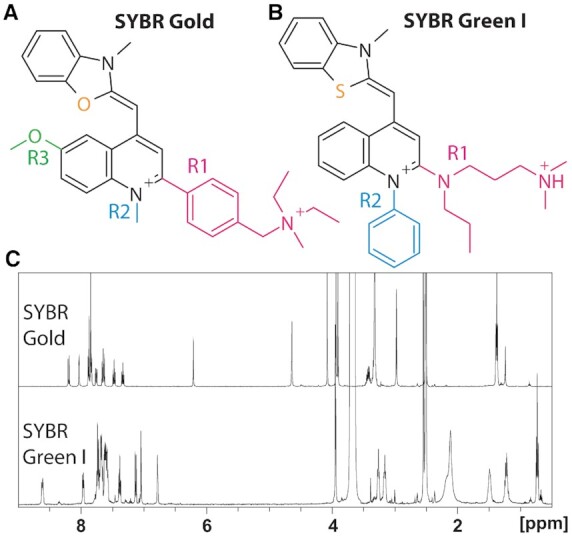
SYBR Gold structure and ^1^H NMR spectra. (**A**) The structure of SYBR Gold as determined by NMR studies and mass spectrometry. For clarity, the side chains are shown in color and named R1, R2, R3. (**B**) The structure of SYBR Green I from (29). The protonation state of the side chain R1 is for aqueous solution near neutral pH. (**C**) ^1^H NMR spectra of SYBR Gold and SYBR Green I recorded in DMSO-d6.

We revealed the molecular structure of SYBR Gold using a combination of NMR and mass spectrometry. All NMR spectra were acquired at 298 K on different NMR spectrometers (Bruker Avance III, with 500/600/800/950 MHz ^1^H frequency, mostly equipped with helium-cooled cryoprobes; Bruker Daltonik, Germany). Besides 1D ^1^H and ^13^C spectra (Figure 1 and Supplementary Figure S2), sets of 2D spectra were acquired to enable the assignment and structure elucidation (2D DQF-COSY, 2D TOCSY, 2D long-range COSY, 2D NOESY, ^1^H,^13^C-DEPT, ^1^H,^13^C–HSQC, ^1^H,^13^C-HMBC, ^1^H,^15^N-HSQC, ^1^H,^15^N-HMBC); sample impurities were identified from DOSY experiments. Starting from the characteristic signal of the cyanine bridge (position 2a', singlet at ca. 6.2 ppm ^1^H/74 ppm ^13^C shift), the whole benzo-X-azole/quinoline scaffold could be assigned from DQF-COSY and ^1^H,^13^C -HMBC correlations. A comparison of the shifts of positions 2a', 3a', 7a', 3a'-CH3 and ^15^N3' (Supplementary Table S2) with the values for SYBR Safe, Thiazole Orange (31) and SYBR Green I (X = S) on one hand, and SYBR Green II and the simulated data for SYBR Gold (X = O) on the other hand (32) unambiguously showed that SYBR Gold must have a benzo-oxazole moiety in its scaffold (which is also in agreement with the MS data). Position and identity of the O-CH_3_ substituent at position 6 of the quinoline moiety were derived from the DQF-COSY and ^1^H,^13^C-HMBC correlations in this part and the characteristic ^1^H and ^13^C shifts of the methyl signals. The structure of the ‘tail’ sidechain (R1 in Figure 1A) was established from ^1^H,^13^C-HMBC correlations between the quinolone moiety and the signals of the 1,4 disubstituted phenyl ring in the tail. The quaternary nitrogen could be identified by ^1^H,^15^N-HMBC correlations between ^15^Nζ and the ^1^H signals of the surrounding positions ϵ, θ and Nζ-CH_3_. After establishing the structure of SYBR Gold, additional long range-COSY and NOESY correlations were studied to see if they would confirm the structure. All correlations found in the experimental NMR spectra were in full agreement with the proposed structure of SYBR Gold. An assignment of the observed NMR peaks is presented in Supplementary Table S2.

Fluorophore standards were run on an ultra-high-performance liquid chromatographic (UHPLC) system coupled to a Bruker timsTOF MS (Bruker Daltonik, Bremen, Germany). Five microliter were injected and separated using a C8 reversed phase column (Ultra C8, 3 μm, 2.1 × 100 mm, Restek GmbH, Bad Homburg, Germany) with 300 μl flow per minute at 60°C. Solvents were water (A) and a mixture (70/30 v/v) of acetonitrile and isopropanol (B), both containing 1% ammonium acetate and 0.1% acetic acid. The gradient started with 1 min at 55% B followed by a ramp to 99% B within 14 min. This was kept constant for 7 min and returned to 55% B with additional 4 min of re-equilibration.

Mass spectra were acquired by otofControl 4.0 in positive MSMS mode from 100 to 1300 *m*/*z* mass range. The most important parameters are set as followed: capillary voltage 4000 V, nebulizer pressure 1.8 bar, nitrogen dry gas 8 l⋅min^–1^ at 200°C, collision energy 70 eV, Collision RD 800 V_pp_ (volt peak to peak). The evaluation was performed by Data Analysis 4.5 provided by Bruker (Bruker Daltonik, Germany).

### DNA constructs

For MT measurements, we use a 7.9-kb DNA construct, prepared as described previously (24). The construct is generated by ligating handles (∼600 bp) with either multiple biotin or multiple digoxigenin moieties fragments to an unmodified central DNA segment 7.9 kb in length. Fluorescence intensity measurements of SYBR Gold are recorded in the presence of linear pBR322 plasmid DNA (NEB), which is produced by restriction of supercoiled circular pBR322 using restriction enzyme EcoRV (NEB) according to the protocol provided by the manufacturer. Completion of the linearization reaction was validated by agarose gel electrophoresis. Absorption, excitation and emission spectra of SYBR Gold are recorded in the presence of lambda phage DNA (NEB) due to large cuvette volumes needed, which was dialyzed against 1× phosphate buffered saline (PBS, consisting of 10 mM phosphate buffer, pH 7.4, with 137 mM NaCl and 2.7 mM KCl; Sigma-Aldrich, USA) prior to use.

### Magnetic tweezers setup

Experiments on DNA are performed on a home-built MT setup described previously (33). Two magnets (5 × 5 × 5 mm^3^; W-05-N50-G, Supermagnete) are placed in a vertical configuration (34,35) on a motorized arm with a translational motor (M-126.PD2 motor with C-863.11-Mercury controller, Physik Instrumente) as well as a rotational motor (C-150.PD motor with C-863.11-Mercury controller, Physik Instrumente) to control the magnets’ rotation and z-position. The flow cell outlet is connected to a pump (ISM832C, Ismatec) for fluid handling. The setup is controlled using a Lab-VIEW software (National Instruments) described by Cnossen *et al.* (36).

Flow cells are built from two coverslips (24 × 60 mm, Carl Roth, Germany). The bottom coverslip was first functionalized using (3-Glycidoxypropyl)trimethoxysilane (abcr GmbH, Germany) and consecutively incubated with 50 μl of a 5000× diluted stock solution of polystyrene beads (Polysciences, USA) in ethanol (Carl Roth, Germany) to serve as reference beads for drift correction. The top coverslip has two openings with a radius of 1 mm for liquid exchange in the flow channel. The bottom and the top coverslip are glued together by a single layer of melted Parafilm (Carl Roth, Germany), precut to form a ∼50 μl channel connecting the inlet and the outlet opening of the flow cell. After the flow cell assembly, one flow cell volume of 100 μg/ml anti-digoxigenin (Roche, Switzerland) in 1× PBS is introduced, and incubated overnight (at least 12 h). Subsequently, the flow cell is rinsed with 1 ml of 1× PBS and then passivated using a commercial passivation mix (BlockAid Blocking Solution, Thermoscientific) for 1 h to minimize non-specific interactions. Unbound material is removed from the flow cell by flushing with 1 ml of 1× PBS.

As magnetic beads we use 1 μm diameter MyOne beads (Life Technologies, USA). The DNA construct is attached to the streptavidin coated beads by incubating 0.5 μl of picomolar DNA stock solution and 2 μl beads in 250 μl PBS for 5 min. Then, the bead-coupled DNA constructs are introduced into the flow cell to bind to the flow cell surface via multiple digoxigenin:anti-digoxigenin bonds.

### Magnetic tweezers measurements

Prior to the experiments, DNA tethered beads are screened for the presence of multiple DNA tethers and torsional constraint by measuring their response to force and torque. To find out whether a magnetic bead is bound by more than one DNA tether to the surface, we introduce negative turns under high tension (*F* = 5 pN). In the case of a single double-stranded DNA tether, high tension prevents the formation of plectonemes at negative linking differences due to DNA melting and consequently, no change in height is observed. In contrast, if a bead is attached via two or more double-stranded DNA molecules, the molecules form braids when the bead is rotated causing a decrease in tether extension. Beads bound by multiple tethers are discarded from further analysis. To evaluate the presence of single strand breaks, positive linking differences are introduced at low force (*F* = 0.4 pN). Overwinding of torsionally constrained DNA leads to the formation of plectonemes, which decrease the tether extension, whereas in nicked DNA tethers, no linking difference can be induced, and the extension remains constant on magnet rotation.

For force-extension analysis, we exclusively examine torsionally unconstrained (nicked) DNA tethers. At first, we calibrate the magnet distance-to-force relation for each bead by recording the transverse fluctuations of the beads at different magnet separations for times ∼100-fold larger than the characteristic time of the system at the corresponding force, and analyze the power spectral density of the transverse fluctuations to quantify the force at each magnet position (37,38). The force-extension relation was then fitted using the worm-like chain (WLC) model (39) to extract the contour length and bending persistence length of the DNA.

To determine the force-extension behavior in the presence of different concentrations of dye, we introduce 200 μl (∼5 cell volumes) of the lowest dye dilution and measure the tether extension at 25 magnet positions corresponding to forces in the range 0.04–5 pN. The measurements are repeated for different dye concentrations in increasing order. After each concentration change, we wait for several minutes to allow for equilibration. We use the previously calibrated force for each bead to construct force-extension curves that are fit using the WLC model to provide the contour length and persistence length as a function of dye concentration.

Rotation curve measurements on torsionally constrained DNA tethers start by introducing 200 μl of 1× PBS in the flow cell using the peristaltic pump, at a flow rate of ∼300 μl min^–1^. While flushing, the magnets are positioned close to the flow cell to establish a force of 6.5 pN. The pulling force helps to prevent the magnetic beads from getting stuck on the surface, and, importantly, the field constrains the free rotation of the bead (40) during flushing, which is a requirement for determining the absolute shifts in DNA twist upon binding. During the actual measurement, the force is kept constant at 0.5 pN. While monitoring the DNA extension, we turn the magnet from negative to positive turns or from positive to negative turns. Then, we introduce 200 μl (∼5 cell volumes) of the lowest dye concentration, wait for several minutes to allow for equilibration, and record another rotation curve measurement at *F* = 0.5 pN. The experimental procedure is repeated for all dye concentrations in increasing order. Processing of the MT data was carried out using custom-written MATLAB routines.

### Absorption, excitation, and emission spectra

Emission spectra on excitation at 495 nm were recorded in PBS in the wavelength range 505–700 nm and excitation spectra for emission at 537 nm were recorded in the wavelength range 400–550 nm, employing a commercial spectrofluorometer and a 1 cm path-length cuvette (Fluoromax Plus; Horiba). While keeping the SYBR Gold concentration constant at 2.5 μM, the DNA concentration in the cuvette was stepwise decreased by replacing a fraction of the DNA solution with the same volume of a buffered solution containing 2.5 μM SYBR Gold. In addition, reference measurements at the same SYBR Gold concentration in the absence of DNA and with only PBS buffer were carried out. Absorbance was recorded using an Evolution™ 201/220 UV–Vis-spectrophotometer in a 1 cm path-length cuvette (ThermoFisher Scientific).

### Fluorescence intensity measurements

Linearized pBR322 plasmid DNA at varying concentrations was pipetted (25 μl) into a well of the well plate reader (Tecan Infinite M1000 PRO; Well plate: corning black polystyrene 384 well microplate with a flat bottom, Sigma-Aldrich, catalogue number: CLS3821). The fluorescence was read out from the bottom of the wells, with the excitation and emission bandwidth set to 5 nm, the gain to 100, the flash frequency to 400 Hz, and the integration time to 20 μs. We chose the excitation and emission wavelengths to be 495 nm and 537 nm (the excitation and emission maxima for SYBR Gold provided by Invitrogen). For control measurements, lambda phage DNA (NEB) at a constant concentration of 3.5 ng/μl and varying SYBR Gold concentration were used and performed otherwise identically.

For fluorescence read-out experiments with a qPCR cycler (CFX96 Touch Real-Time PCR Detection System, Bio Rad) again linearized pBR322 DNA was used and dilution series were filled into low-profile PCR tubes (Bio Rad, product ID: TLS-0851), which were closed with flat, optical, ultra-clear caps (Bio Rad, product ID: TCS-0803). We used the channel with absorption and emission wavelengths of 494 and 518 nm, respectively, which are the closest match to those of SYBR Gold and read out the fluorescence intensities at 24°C from the top.

For the gel electrophoresis we added Gel Loading Dye Purple (6×) (NEB) to pBR322 DNA. We used 1%-agarose (Carl Roth) gels and TAE buffer (40 mM Tris, 20 mM acetic acid, and 1 mM EDTA, pH 8.6). The gels were run for 120 min at 75 V. Afterwards the gel was removed from the gel box and placed for 20 minutes in 100 ml of 1.24, 3.1 or 6.2 μM SYBR Gold in TAE buffer, respectively, for staining. Subsequently, the gel was de-stained in TAE buffer for 15 min at room temperature. The gels were then visualized using a Gel Doc XR+ system (Biorad).

### Fluorescence lifetime measurements

Fluorescence lifetime measurements were carried out on a homebuilt apparatus for time-correlated single-photon counting (TCSPC). Pulsed excitation was at 485 nm (PicoQuant LDH-D-C-485, controller: PicoQuant PDL 828 ‘Sepia II’, 20 or 13.33 MHz laser repetition rate) to excite the sample cuvette. A lambda-half waveplate (Laser Components ACWP-450-650-10-2-R30 AR/AR, Ø1’) and a linear polarizer (Edmund Optics POLARIZER 25.4 mm DIA UNMTD) were used to rotate and adjust the excitation polarization to vertically polarized light. Light emitted by the sample after excitation was filtered for polarization with a second linear polarizer (Edmund Optics ULTRA BB WIREGRID POL 25RND MTD) under magic angle condition (54.7°) with respect to the vertical axis. The emission polarizer was mounted inside a 3D custom-printed chassis housing with automated rotation mount (Thorlabs CRM1/M, Reichelt GRABIT SERVO), which was controlled by a processing unit (Arduino Mega 2560) for automated rotation of the emission polarizer. A lens (Thorlabs AC254-110-A) was used for higher collection efficiencies in combination with filters (AHF 488 long pass filter BLP01-488-R-25 and notch filter ZET488NF) for removal of scattered laser light before the avalanche photodiode used for detection (Excelitas SPCM-AQRH-34). Data were recorded by a TCSPC unit (PicoQuant HydraHarp 400, 16 ps TCSPC time resolution) with a commercial control and evaluation software provided by the supplier (PicoQuant SymPho Time 64). Further details are as described (41).

We diluted lambda phage DNA (NEB), dialyzed overnight against PBS, and SYBR Gold to the specified dye concentrations (0.062–124 μM) and analyzed the sample in a 10 × 2 mm^2^ cuvette (Perkin Elmer UV/VIS Spectroscopy Cell B0631122) for 5 min for each condition. The excitation laser power was 1.5 or 15 μW in order to operate in an optimal range with the photon detection rates between 20 and 80 kHz. Additionally, we measured the instrument response function (IRF) by replacement of the cuvette with a silver mirror to direct the laser beam directly onto the APD chip, which gave the best IRF quality (scattering based approaches with e.g. Ludox suspension resulted in lower quality IRF).

For the fluorescence lifetime evaluation, we used the *n*-exponential reconvolution fit that is implemented in the measurement software SymPho Time 64. The optimization uses a maximum likelihood estimator, where the fluorescence signal $I( t )$ is described as the convolution of the IRF $IRF( {t - {t_{\textrm{off}}}} )$ with a triple exponential decay (42):

(1) $$\begin{eqnarray*}I\ (t) &=& \ IRF ( {t - {t_{\textrm{off}}}})*\left({A_{\textrm{mm}}}{e^{-\frac{t}{\tau_{\textrm{mm}}}}}+{A_{\textrm{long}}}{e^{-\frac{t}{\tau_{\textrm{long}}}}}+{A_{\textrm{short}}}{e^{-\frac{t}{\tau_{\textrm{short}}}}}\right) +\textrm{bkg}\nonumber\\ &&\end{eqnarray*}$$

In this formula, the first exponential component ${A_{\textrm{mm}}}{e^{ - \frac{t}{{{\tau _{\textrm{mm}}}}}}}$ accounts for a small mismatch between measured IRF (with a mirror) and the ‘real’ IRF in the cuvette measurements. This mismatch was compensated by including a fast decay component in the fitting procedure, with a fixed decay time ${\tau _{\textrm{mm}}} = \ 50\ {\rm{ps}}$, which is 1–2 orders of magnitude faster than the lifetime components of the fluorophore. The fluorophore lifetime was described with two exponential decays with lifetimes ${\tau _{\textrm{long}}}$ and ${\tau _{\textrm{short}}}$, accounting for a fast component, which becomes relevant at high dye concentrations, in addition to the dominant slow lifetime. The reported lifetimes in the main text are amplitude-weighted averages ${\tau _{\textrm{dec}}} = \frac{{{A_{\textrm{long}}}\ {\tau _{\textrm{long}}} + {A_{\textrm{short}}}\ {\tau _{\textrm{short}}}}}{{{A_{\textrm{long}}} + {A_{\textrm{short}}}}}$ of the two lifetime components ${\tau _{\textrm{long}}}$ and ${\tau _{\textrm{short}}}$. The errors for ${\tau _{\textrm{dec}}}$ are derived from the fit uncertainty of all parameter based on standard error propagation rules for non-independent combined quantities.

### Binding models: McGhee-von Hippel model and DNA concentration effects

In order to describe the binding of SYBR Gold and SYBR Green I to double-stranded DNA in our single-molecule tweezers assays, where the DNA concentration is very low and, therefore, the free and total ligand concentrations approximately equal, we used the McGhee-von Hippel model of ligand-substrate binding (43) for the fractional number of molecules bound per base pair, ${\rm{\gamma }}$:

(2) $$\begin{equation*}{\rm{\gamma \ }} = \frac{{{c_{\textrm{free}}}}}{{{K_d}}}\ \cdot \frac{{{{\left( {1 - n \cdot \gamma } \right)}^n}}}{{{{\left( {1 - n \cdot \gamma + \gamma } \right)}^{n - 1}}}}\end{equation*}$$

where ${c_{\textrm{free}}}$ (≈ ${c_{\textrm{total}}}$ under these conditions) is the free ligand concentration, ${K_d}$ is the binding (dissociation) constant (in M) and $n$ is the binding site size (in base pairs). ${\rm{\gamma }}$ was determined from the DNA contour length change with increasing ligand concentration determined in stretching experiments on rotationally unconstrained DNA as

(3) $$\begin{equation*}\gamma \ = \frac{{{L_c}\left( {{c_{\textrm{free}}}} \right) - {L_c}\left( 0 \right)}}{{\Delta z\ \cdot \ {N_{bp}}}}\ \end{equation*}$$

where ${L_c}( {{c_{\textrm{free}}}} )$ is the DNA contour length at a certain ligand concentration, $\Delta z$ is the increase in DNA contour length per ligand bound, and ${N_{bp}}$ is the number of base pairs of the DNA (${N_{bp}}$ = 7922 for our DNA constructs). We used a fixed value for the DNA contour length increase per dye binding event $\Delta z$ = 0.34 nm, as was suggested previously for intercalators (3,15,24,44).

From bulk experiments using a plate reader, qPCR cycler, or a gel imager we determined the fluorescence intensity $I$ as a function of DNA concentration ${c_{\textrm{DNA}}}$ and dye concentrations ${c_{\textrm{total}}}$. For bulk measurements, the assumption ${c_{\textrm{free}}} \approx {c_{\textrm{total}}}$ often does not hold, as the free ligand concentration is on the order of the bound ligand concentration and both the finite dye and DNA concentrations need to be taken into account. Therefore, we rewrote Equation (2) in terms of ${c_{\textrm{total}}}$, which is experimentally known, by expressing the total ligand concentration as the sum of free ligand concentration and bound ligand concentration:

(4) $$\begin{equation*}{c_{\textrm{total}}} = {c_{\textrm{free}}}\ + {c_{\textrm{bound}}}\end{equation*}$$

We define

(5) $$\begin{equation*}\gamma \ = \frac{{{c_{\textrm{bound}}}}}{{{c_{\textrm{DNA}}}}}\ \end{equation*}$$

with ${c_{\textrm{DNA}}}$ being the total DNA concentration which is also experimentally known. Subsequently, we combined Equations (4) and (5) and inserted them into Equation (2) to obtain:

(6) $$\begin{equation*}\frac{{{c_{\textrm{bound}}}}}{{{c_{\textrm{DNA}}}}} = \frac{{\left( {{c_{\textrm{total}}} - {c_{\textrm{bound}}}} \right)}}{{{K_d}}}\ \cdot \frac{{{{\left( {1 - n \cdot \frac{{{c_{\textrm{bound}}}}}{{{c_{\textrm{DNA}}}}}} \right)}^n}}}{{{{\left( {1 - n \cdot \frac{{{c_{\textrm{bound}}}}}{{{c_{\textrm{DNA}}}}} + \frac{{{c_{\textrm{bound}}}}}{{{c_{\textrm{DNA}}}}}} \right)}^{n - 1}}}}\end{equation*}$$

We call Equation (6) the McGhee-von Hippel model for finite DNA concentrations.

To fit our fluorescence intensity data with the McGhee-von Hippel model for finite DNA concentrations, we assume that the fluorescence intensity is proportional to the concentration of bound SYBR Gold molecules

(7) $$\begin{equation*}I\ = \frac{1}{\alpha }\ \cdot {c_{\textrm{bound}}}\end{equation*}$$

where $\alpha$ is a proportionality constant that we treated as a fitting parameter. For SYBR Gold the fluorescence intensity is reported to increase by more than a factor of 1000 upon binding to double-stranded DNA (1,2), consistent with our measurements (Supplementary Figure S3, compare the dye only spectrum in the inset with the spectrum in the presence of the highest DNA concentration). Experimentally, we found that measurements in absence of DNA give much lower fluorescence intensity than measurements even at low DNA concentrations (Supplementary Figure S3), therefore, we neglected fluorescence contributions from the free dye. We substituted the concentration of bound dye molecules ${c_{bound}}$ in Equation (6) by $\alpha$*⋅ I* to obtain the final equation that can be fit to the fluorescence intensity data:

(8) $$\begin{equation*}\frac{{\alpha \cdot I}}{{{c_{\textrm{DNA}}}}} = \frac{{\left( {{c_{\textrm{total}}} - \alpha \cdot I} \right)}}{{{K_d}}}\ \cdot \frac{{{{\left( {1 - n \cdot \frac{{\alpha \cdot I}}{{{c_{\textrm{DNA}}}}}} \right)}^n}}}{{{{\left( {1 - n \cdot \frac{{\alpha \cdot I}}{{{c_{\textrm{DNA}}}}} + \frac{{\alpha \cdot I}}{{{c_{\textrm{DNA}}}}}} \right)}^{n - 1}}}}\end{equation*}$$

In Equation (8), the total ligand concentration ${c_{\textrm{total}}}$ as well as the total DNA concentration ${c_{\textrm{DNA}}}$ are experimentally known, the fluorescence intensity *I* is measured, and the dissociation constant ${K_d}$, the binding site size $n$, and the proportionality factor $\alpha$ are fitting parameters. For the gel imager data, we need to take into account that the concentration in the staining solution is not equal to the final concentration in the gel, due to dilution by the gel volume, incomplete penetration of the dye into the gel, and the final de-staining step. We, therefore, rescale the SYBR Gold concentrations for all gel data with a single global dilution factor, which we determine to be ≈0.1.

## RESULTS

To investigate the binding mode of SYBR Gold to DNA and to quantitatively monitor the resulting changes in DNA properties and fluorescence increase upon SYBR Gold binding, we use different, complementary techniques. First, we determined the molecular structure of SYBR Gold and its extinction coefficient to calibrate the concentration of the stock solution. Then, in a first series of experiments, we use MT micromanipulation to monitor binding and examine the effects of SYBR Gold and of the related dye SYBR Green I on the structural and mechanical properties of DNA under controlled stretching forces and degrees of supercoiling. In a second set of experiments, we monitor SYBR Gold fluorescence in the presence of various concentrations of dye and DNA via fluorescence spectroscopy.

### SYBR Gold structure determination

Using NMR analysis and mass spectrometry, we determined the molecular structure of SYBR Gold to be [2-(4-{[diethyl(methyl)ammonio]methyl}phenyl)-6-methoxy-1-methyl-4-{[(2Z)-3-methyl-1,3-benzoxazol-2-ylidene]methyl}quinolin-1-ium] (Figure 1A). The structure assignment used 1D ^1^H and ^13^C spectra (Figure 1C and Supplementary Figure S2) as well as several sets of 2D correlation spectra (see ‘Structure determination’ in Materials and Methods and Supplementary Table S1). The main population in the mass spectrum is at *m*/*z* = 247.6, in excellent agreement with the prediction from the structure of *m*/*z* = 247.5 and charge +2, corresponding to a molecular mass of SYBR Gold of 495.2 Da (Supplementary Figure S4). Minor populations are found at *m*/*z* = 408.2 and around 480 and very likely correspond to fragments of the molecule with *z* = +1 after dissociation of one or several methyl groups at the side chain amine. The SYBR Gold core structure consists of benzoxazole and quinoline heterocycles connected by a monomethine group. It is identical to the core of SYBR Green II and similar to the core structures of SYBR Green I, Pico Green, and SYBR Safe, which feature a benzothiazole instead of the benzoxazole (29) (Figure 1B). In addition to O versus S in the core, the main differences between the SYBR family dyes are in the side chain substitutions of the quinolone moiety (labeled R1–R3 in Figure 1A, B). SYBR Gold has a charge of +2 in aqueous solution around neutral pH as well as in DMSO, in contrast to SYBR Green I, which is divalent in aqueous solution, but monovalent in DMSO.

### SYBR Gold and SYBR Green I lengthen the DNA contour

**Figure 2.**
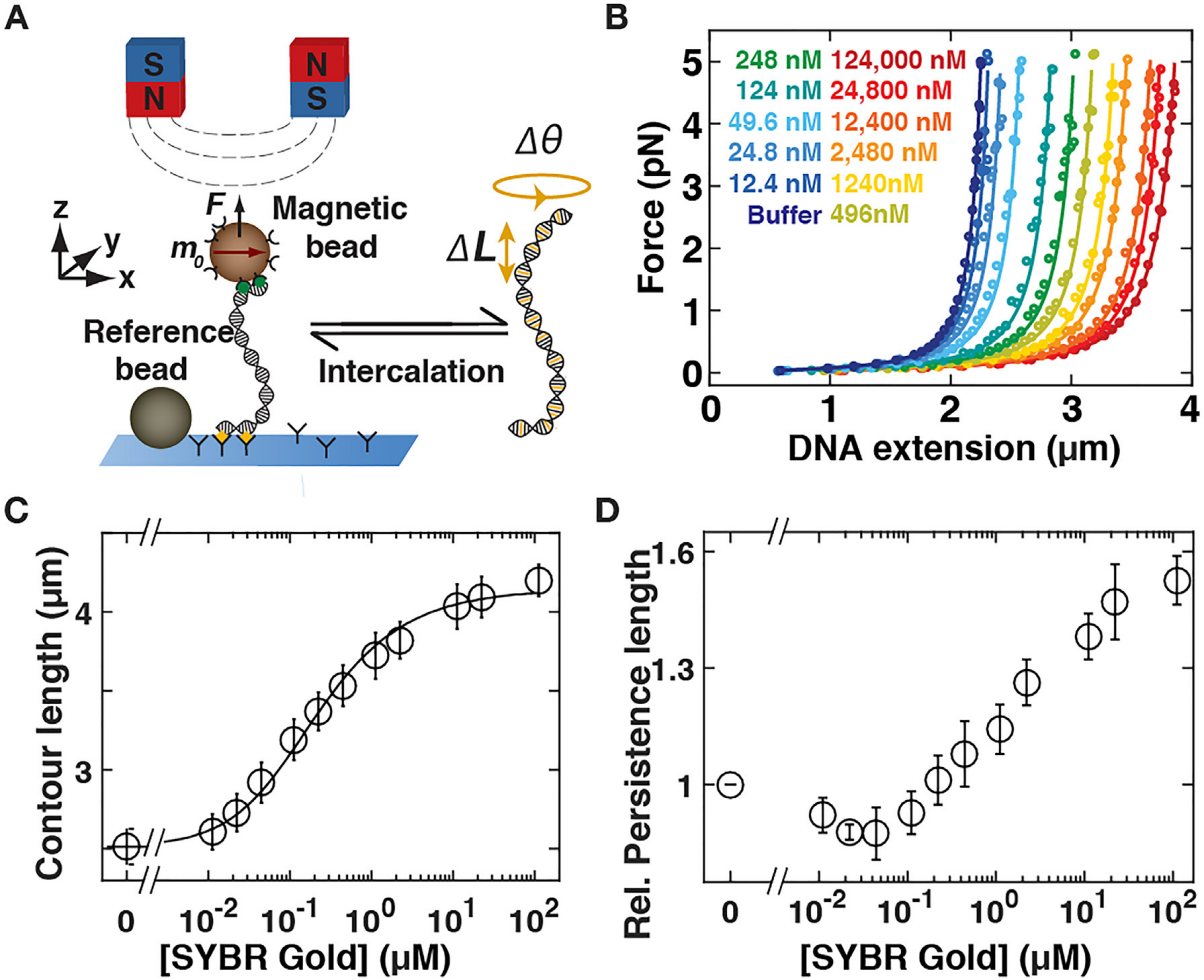
Effects of SYBR Gold on the force-extension behavior of DNA. (**A**) Schematic of magnetic tweezers. DNA molecules are tethered between a surface and magnetic beads in a flow cell. Permanent magnets above the flow cell enable the application of stretching forces and torques, respectively. Upon intercalation, the DNA molecules lengthen and change their equilibrium twist. (**B**) Force-extension curves for 7.9-kb DNA in the presence of increasing concentrations of SYBR Gold (increasing concentrations from blue to red indicated in the figure legend). Symbols are raw data, lines are fits of the WLC model. A systematic increase the DNA extension with increasing SYBR Gold concentration is apparent. (**C**) DNA contour length determined from fits of the WLC model as a function of SYBR Gold concentration. The black line is a fit to the McGhee–von Hippel model (reduced *χ*^2^ = 0.18; see main text for details), with a dissociation constant ${K_d}$ = (2.73 ± 0.26) × 10^–7^ M and a binding site size *n* = 1.67 ± 0.04. (**D**) DNA bending persistence length from WLC fits measured as a function of the dye concentration, normalized to the bending persistence length measured for bare DNA, indicating that the persistence length increases with increasing amount of SYBR Gold. Data points and error bars in panels C and D are the mean and standard deviation from at least ten independent measurements. In panel B one typical experiment is shown for clarity.

To determine the binding mode and influence on DNA structure of SYBR Gold and SYBR Green I we performed MT experiments at varying concentrations using a custom-built multiplexed MT setup (Figure 2A and Materials and Methods). To assess to what extent SYBR Gold or SYBR Green I binding lengthens the DNA contour, we first performed DNA stretching experiments in the presence of increasing concentrations of dye on torsionally relaxed (nicked) DNA. We focused on the force regime <5 pN for our force–extension measurements: in this regime the DNA extension is well described by the worm-like chain (WLC) model of entropic stretching elasticity (45) and we can neglect enthalpic contributions to stretching and the force-dependence of intercalative binding (3,15,46). In the absence of dye the response of DNA to force shows the characteristic response of entropic stretching elasticity (Figure 2B, dark blue circles). A fit to the WLC model (39) (Figure 2B, dark blue line) yields a contour length ${L_C}$ = (2.5 ± 0.1) μm (mean and standard deviation from ten independent measurements), in close agreement with the expected crystallographic length of 2.6 μm expected for a 7.9-kb DNA. We then examined the force-extension response of torsionally unconstrained DNA at increasing concentrations of SYBR Gold (Figure 2B, curves from blue to red) and SYBR Green I (Supplementary Figure S5A, curves from blue to red). Fitting the WLC model to the force-extension data demonstrates that the contour length of the molecule systematically increases with increasing dye concentration: compared to the contour length of bare DNA, the contour length of DNA in the presence of ∼10 μM SYBR Gold or SYBR Green I is increased by a factor of 1.7. A contour length increase upon binding by ∼1.7-fold is in line with previous force spectroscopy measurements on SYBR Gold (3) and indicative of an intercalative binding mode (9,15,24). An increase of the contour length by ∼1.7-fold is very unlikely to result from minor or major groove binding and is similar to the length increase in the DNA overstretching transition at much higher forces, which both have been suggested to be limited by the dihedral sugar angle in the DNA backbone (19). The increase in the contour length follows a binding curve behavior and saturates at concentrations ≥2.5 μM (Figure 2C and Supplementary Figure S5C). To quantitatively determine binding parameters from the mechanical response of DNA, we employ the McGhee-von Hippel model (Equation 1 in Materials and Methods) (43). The fractional number of bound dye molecules is computed from the fitted contour length ${L_C}( c )$ at a given concentration *c* by Equation (2). We use a value for the length increase per dye molecule bound of $\Delta z$ = 0.34 nm, typical for intercalation (3,15,24,44). Fitting the McGhee–von Hippel model to the contour length data determined from our force-extension measurements, we find the dissociation constant ${K_d}$ = (2.73 ± 0.26) × 10^–7^ M and a binding site size $n$ = 1.67 ± 0.04 for SYBR Gold and very similar values of ${K_d}$ = (3.33 ± 0.7) × 10^–7^ M and $n$ = 1.77 ± 0.06 for SYBR Green I. Errors are obtained from a ‘bootstrapping’ procedure (47) by generating 1000 synthetic data sets from the experimental data and computing the standard deviation over repeated McGhee–von Hippel fits. The value of the binding site size $n$ can be interpreted as SYBR Gold or SYBR Green I intercalation at saturation occurring slightly more than at every other basepair (48), similar to other monointercalators with similar structures (3,15,24,49). In addition to an increase in DNA contour length upon SYBR Gold binding, the effective bending rigidity is also changing (Figure 2D). We found ${L_P}$= (38 ± 4) nm in the absence of dye. This value is in agreement though slightly lower than reported data for DNA persistence length in PBS (24). With increasing SYBR Gold concentration, the persistence length initially stays constant or decreases very slightly up to a concentration of ∼0.16 μM and then significantly increases at higher dye concentrations (Figure 2D). In comparison to the persistence length of bare DNA, the persistence length of DNA in the presence of ∼10 μM SYBR Gold increases by a factor of 1.55. In contrast, for SYBR Green I we observe no systematic change in *L_P_* (Supplementary Figure S5D). Previous studies of intercalators have reported both decreases in effective bending stiffness, e.g. for ethidium bromide, PicoGreen, or a Ru(II) metallo-intercalator (24,49,50), but also constant or increasing stiffness, e.g. for the bis-intercalators YOYO and TOTO (26,51,52). We speculate that the observed increase of *L_P_* upon SYBR Gold binding might be due to steric interactions of the bulky phenyl group in the side chain at position R1, which is missing in the otherwise similar dyes PicoGreen and SYBR Green I.

### SYBR Gold and SYBR Green I untwist DNA

**Figure 3.**
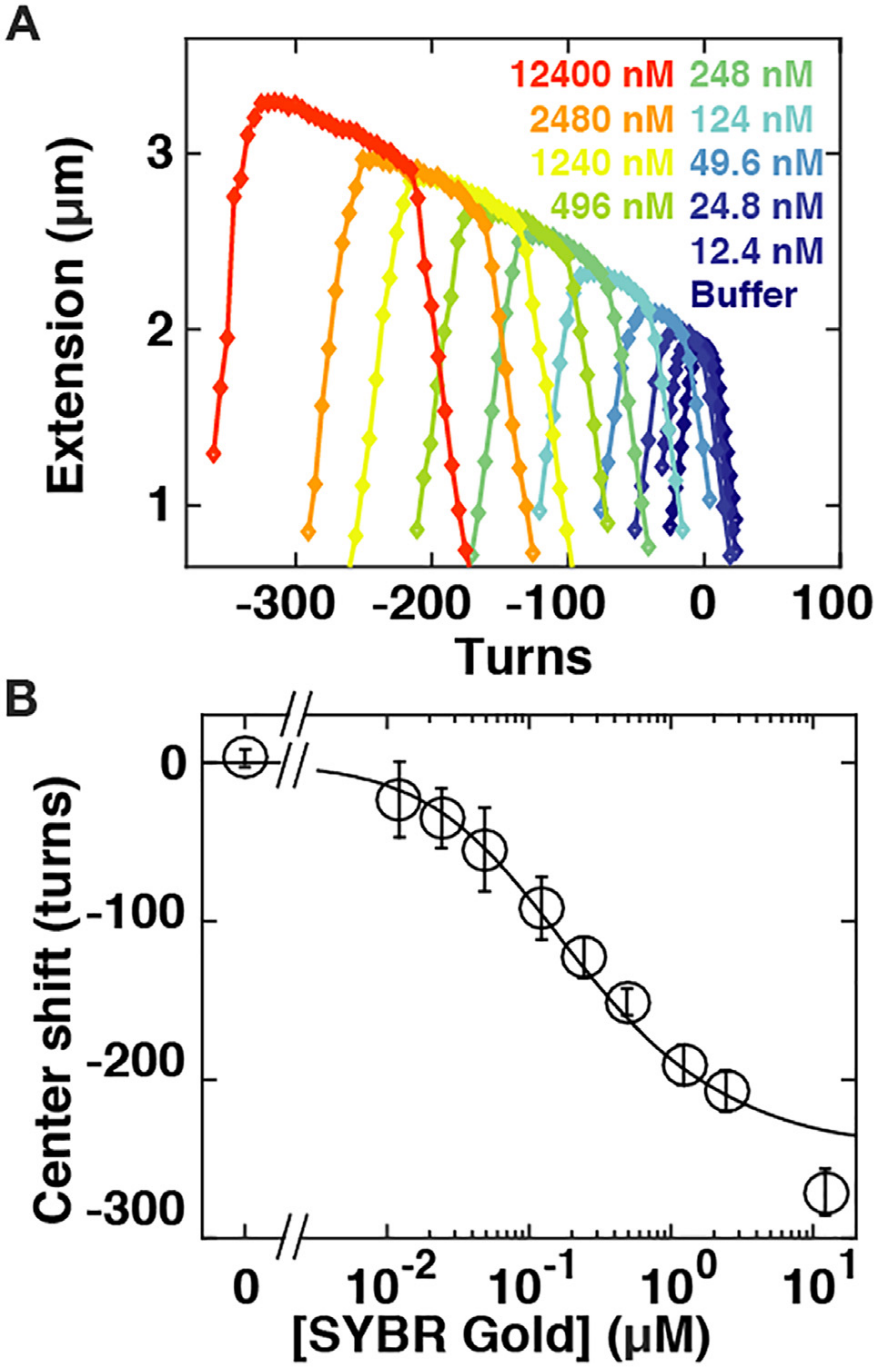
Effects of SYBR Gold on DNA twist. (**A**) Rotation-extension curves for 7.9 kb DNA at *F* = 0.5 pN in the presence of increasing concentrations of SYBR Gold. The SYBR Gold concentrations are (from blue to red) 0, 12.4, 24.8, 49.6, 124, 248, 392, 1240, 2480, 12 400 nM. With increasing concentrations of SYBR Gold the rotation curves shift to negative turns; the DNA length at the center of the curves increases; and the rotation-extension curves broaden. (**B**) Quantification of the shift in the center position of the rotation-extension curves as a function of the SYBR Gold concentration. The center positions were determined from fitting slopes in the positive and negative plectonemic regime and by computing the intersection of the two slopes. The black line is a fit of the McGhee–von Hippel model (reduced *χ*^2^ = 2.9; see main text for details), with the dissociation constant *K*_d_ and binding site size *n* set to the values determined from the force extension data (Figure 2) and the unwinding angle per SYBR Gold intercalation determined from the fit to be *Δθ* = 19.1° ± 0.7°. Data points and error bars in panel B are the mean and standard deviation from at least 14 independent measurements. In panel A one typical experiment is shown for clarity.

Force-extension measurements of torsionally unconstrained DNA in the presence of different concentrations strongly suggest that SYBR Gold and SYBR Green I both bind DNA in an intercalative binding mode. To further investigate their binding to DNA, we probe the effects of varying concentrations of dye on DNA twist using rotationally constrained DNA molecules (24). We control the DNA linking number $Lk$ by rotation of the magnets and monitor DNA extension as a function of applied magnet turns at a low constant force $F\ = \ 0.5\ {\rm{pN}}$ (Figure 3A). In the absence of dye, we observe the characteristic response of bare DNA (Figure 3A, dark blue data): Initially the change in turns ($\Delta Lk$) leads to elastic twist deformations of DNA that minimally affect the tether extension. Subsequently, the DNA buckles to form plectonemic supercoils. In the plectonemic regime, a further increase of $\Delta Lk$ results in a linear reduction of its end-to-end extension. For measurements at low forces (here $F\ = \ 0.5\ {\rm{pN}}$), the response of the DNA is symmetric about $L{k_0}$ (i.e. zero applied turns, corresponding to a torsionally relaxed molecule): if we introduce positive or negative turns the DNA forms positive or negative plectonemic supercoils, respectively. Throughout, we use the number of applied turns where the DNA tether is torsionally relaxed in the absence of added SYBR Gold, i.e. $Lk\ = L{k_0}$, as a reference. The point corresponding to $Lk\ = L{k_0}$ was determined as the midpoint of the symmetric rotation curve (Supplementary Figure S6A). Addition of increasing concentration of SYBR Gold or SYBR Green I leads to dramatic changes in the shape and position of the extension vs. applied turns curves (Figure 3A and Supplementary Figure S5B, increasing dye concentration from blue to red). There are four effects that become increasingly pronounced with increasing dye concentration: (i) an overall increase of the DNA tether length, (ii) a shift of the centers of the curves towards negative turns, (iii) a broadening of the curves, i.e. an extension of the pre-buckling regime and (iv) a negative slope of extension vs. turns in the pre-buckling regime. In the following, we will discuss each of these observations:

- The increase in the extension with increasing SYBR Gold or SYBR Green I concentration is readily understood from the force-extension measurements discussed in the previous section. The center of the rotation-extension curves corresponds to torsionally relaxed DNA for which a systematic length increase with increasing SYBR Gold concentration by up to ∼1.7-fold at saturating dye concentrations was observed in the force-extension measurements (Figure 2C).
- The shift in center position of the rotation-extension curves with increasing dye concentration compared to bare DNA is indicative of DNA unwinding upon binding, which is again consistent with intercalation. We can understand the shift following the addition of SYBR Gold or SYBR Green I by considering that our DNA molecules are torsionally constrained ($Lk$ is a topological constant). If dye binding causes a change in the DNA $Tw$, compensatory changes in $Wr$ must occur, which, in turn, result in a reduction of the DNA end-to-end extension due to the formation of plectonemes. We quantified the shift in the extension curves by linearly fitting the extension versus applied turns response in the plectonemic regime for both positive and negative plectonemes and determine the center of the curve as the intersection of the two slopes. Plotting the shift in the rotation curve centers as a function of the SYBR Gold concentration (Figure 3B), we again obtain a binding curve behavior. We fit the center shifts by $\Delta Tw( {[ {dye} ]} )\ = \ \gamma \cdot Nbp \cdot \Delta \theta$, where we use $\gamma$ as computed from the force extension measurements (Figure 2C). The change in DNA twist per binding event $\Delta \theta$ is treated as a fitting parameter. The resulting fit (Figure 3B, solid line, and Supplementary Figure S5E) gives $\Delta \theta$ = 19.1° ± 0.7° for SYBR Gold and $\Delta \theta$ = 19.3° ± 1.3° for SYBR Green I. The errors were computed as the standard deviation over fits to 1000 synthetic bootstrap data sets. The fitted values for $\Delta \theta$ are comparable to values found for other intercalators using magnetic tweezers manipulation, for example 27.3° ± 1° for ethidium bromide (24,53) or 21° ± 14° for Pico Green (49) (Supplementary Table S3). The very similar values for $\Delta \theta$ measured for SYBR Gold and SYBR Green I suggest that the side chains R1–R3 (Figure 1A, B), where SYBR Gold and SYBR Green I differ, do not significantly contribute to the intercalation geometry. In contrast, the phenanthridine moiety of ethidium appears to lead to greater unwinding of the DNA helix compared SYBR Gold or SYBR Green I. The slopes in the plectonemic regime at $F\ = \ 0.5\ {\rm{pN}}$ are the same, within experimental error, for positive and negative supercoils and also do not change, again within error, with increasing concentrations of SYBR Gold or SYBR Green I (Supplementary Figure S6B and S5F).
- and (iv) Broadening of the rotation curves and a slope in the pre-buckling regime for SYBR Gold and SYBR Green I (Figure 3A, Supplementary Figure S6C for SYBR Gold, and Supplementary Figure S5B, Supplementary Figure S5G for SYBR Green I) are similar to what has been observed for other intercalators, notably ethidium bromide (24,53), PicoGreen (49) and the bis-intercalator YOYO-1 (27). The two effects can be understood from the properties of the DNA tethers and how they are changed upon SYBR Gold intercalation. Fundamentally, a molecule buckles if the energy required to form a plectoneme becomes less than the twist energy stored in the chain induced by adding turns to the molecule. For naked DNA in the pre-buckling regime, the torque builds up as $\Gamma \ = \ ( {{k_B}T \cdot C/{L_C}} ) \cdot 2\pi \cdot \Delta Lk$, where ${k_B}$ is the Boltzmann constant, $T$ the absolute temperature, and $C$ the torsional persistence length. Buckling occurs once the built-up torque reaches the critical torque for buckling ${\Gamma _{buck}}$, which increases with increasing bending persistence length, approximately (54,55) as ${\Gamma _{buck}} \approx {( {{k_B}T \cdot {L_P} \cdot F} )^{1/2}}$. Part of the broadening of the rotation curves can, therefore, likely be explained by changes in the mechanical properties of DNA in the presence of SYBR Gold. The observed increase in bending persistence length (Figure 2D) will tend to increase the number of turns required for buckling (yet only by 1.6^1/2^ ≈ 1.25-fold); similarly, a decrease in the torsional persistence length, as has been observed for ethidium bromide upon intercalation (56), would increase the number of turns required for buckling and broaden the curves (24). However, in addition to changing mechanical properties in the presence of intercalation, torque-dependent intercalation will also contribute to the broadening of the rotation curves. The extension is maximal not at the center of the rotation-extension curve, as is the case for bare DNA, but at negative turns just before buckling, due to the overall slope in the pre-buckling regime. The dependence of tether extension on the number of applied turns at a given force and SYBR Gold or SYBR Green I concentration suggests that the application of a negative torque increases binding, whereas the application of a positive torque hinders it, which is to be expected from Le Chatelier's principle as intercalation unwinds the DNA helix. We note that the slopes of the plateaus in the pre-buckling regime corresponded roughly to the slope of the curve connecting the center position of the rotation–extension curves, similar to what has been observed for ethidium bromide (53). The center positions of the rotation-extension curves are given by the coupling between DNA elongation and untwisting upon intercalation.

The observation that the slope in the pre-buckling plateaus matches the slope of the line connecting the rotation curve centers suggests that upon twisting dye-bound DNA, the applied turns are predominantly absorbed by torque-induced intercalation, again suggesting an important role of torque-dependent binding, due to the unwinding of the helix upon intercalation. In conclusion, the results of our DNA micromanipulation experiments reveal that SYBR Gold and SYBR Green I binding to DNA systematically lengthens, by up to ∼1.7-fold, and unwinds DNA, by 19.1° ± 0.7° and 19.3° ± 1.3°, respectively, per binding event, strongly suggesting intercalative binding for both dyes.

### SYBR Gold fluorescence enhancement by DNA intercalation

**Figure 4.**
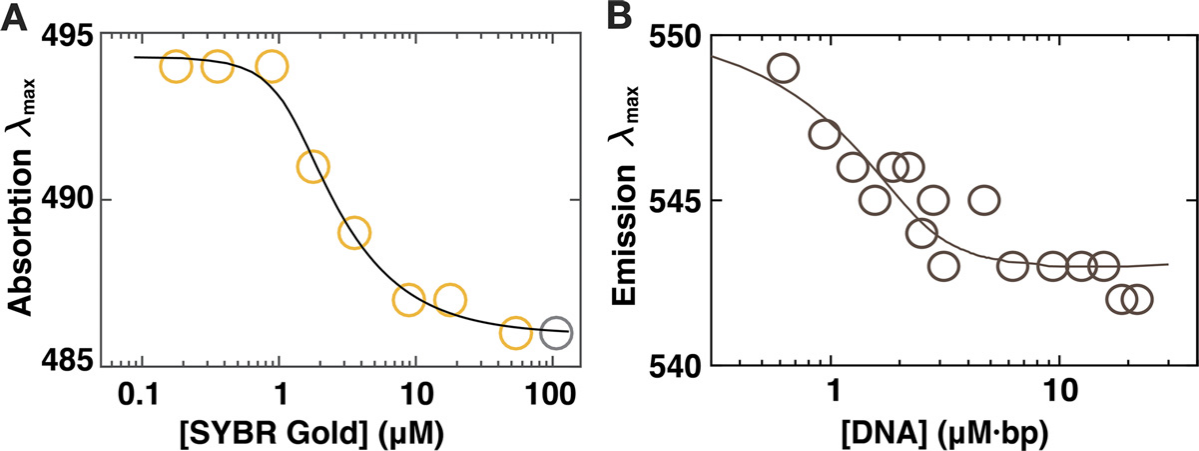
Shifts in SYBR Gold absorbance and emission spectra. (**A**) Position of the absorbance maxima of the SYBR Gold absorbance spectra (Supplementary Figure S3A) as a function of the SYBR Gold concentration (circles). The peak of the spectrum at the highest concentration is noisy, as the dynamic range of the instrument is approached, thus the point from this spectrum is included but greyed out. The solid line is a fit to the finite concentration McGhee-von Hippel model with the wavelengths for maximum absorbance of free *λ*_max,free_ and intercalated *λ*_max,bound_ SYBR Gold as the only fitting parameter (see main text and Equation 9). From the fit we find *λ*_max,free_ = 486 nm and *λ*_max,intercalated_ = 494 nm. (**B**) Position of the emission maxima at constant SYBR Gold concentration (2.5 μM) and varying DNA concentrations. The data are well described by the model in Equation 9, with binding parameters *K_d_* and *n* fixed to the values determined from the MT measurements (analogous to panel A). From the fit we find for *λ*_max,free_ = 550 nm and *λ*_max,bound_ = 543 nm for emission.

To relate the SYBR Gold binding behavior revealed by the MT measurements to SYBR Gold fluorescence, we first determined absorption, excitation, and emission spectra (Supplementary Figure S3A, B) of the dye in the presence of DNA. The absorption spectra show a systematic increase of absorbance with SYBR Gold concentration, at fixed DNA concentration, with a peak around 490 nm (Supplementary Figure S3A) and a second absorption band <300 nm, consistent with previous measurements (1,2). The position of the main visible absorbance peak shifts to shorter wavelength with increasing SYBR Gold concentrations at constant DNA concentration (Figure 4A). The position of the peak is well described by a model (Figure 4A, solid line) that assumes fixed absorbance peak wavelengths λ_max,free_ and λ_max,bound_ for free and bound SYBR Gold, respectively, and a linear superposition of the form

(9) $$\begin{eqnarray*}{\lambda _{\textrm{max}}}\left( {{c_{\textrm{total}}},{c_{\textrm{DNA}}}} \right)\ = \ \frac{{{c_{\textrm{free}}}}}{{{c_{\textrm{total}}}}} \cdot {\lambda _{\textrm{max},\ \textrm{free}}} + \frac{{{c_{\textrm{bound}}}}}{{{c_{\textrm{total}}}}} \cdot {\lambda _{\textrm{max},\ \textrm{bound}}}\nonumber\\ \end{eqnarray*}$$

The free and bound SYBR Gold concentrations are computed from the finite concentration McGhee-von Hippel model (Equation 6), using the *K*_d_ and *n* values determined from the MT force-extension measurements (Figure 2). The absorbance peak wavelengths are determined from a fit as λ_max,free_ = 486 nm and λ_max,bound_ = 494 nm, very close to the previously reported values of 487 and 495 or 496 nm for free and bound SYBR Gold, respectively (1,2) and in excellent agreement with the position of the maximum of 486 nm determined in the absence of DNA (Supplementary Figure S1). The absorbance increases linearly with increasing SYBR Gold concentration (Supplementary Figure S3C), which suggests that absorption does not depend on the dye being intercalated or in free solution (since the DNA concentration is fixed for the data set in Supplementary Figure S3C). The slope of the linear dependence agrees well with the value for ϵ_486 nm_ used in the concentration determination using a different instrument (Supplementary Figure S3C and Materials and Methods), which represents a consistency check.

The excitation and emission spectra exhibit single peaks in the range probed (Supplementary Figure S3B). While the position of the excitation peak at 496 nm does not shift significantly upon binding (Supplementary Figure S3D), we observe a systematic shift for the emission peak (Figure 4B). The emission data are well described by the model that was fit to the absorbance data (Equation 9), using again the finite concentration McGhee-von Hippel model with *K*_d_ and *n* fixed to the values determined from the MT measurements and fitting *λ*_max,free_ and *λ*_max,bound_ for emission. We find *λ*_max,free_ = 550 nm and *λ*_max,bound_ = 543 nm for emission, similar to, but slightly higher than the values reported previously (1,2). The difference is likely within experimental error and we note that the magnitude of the shift is very similar to what was previously reported. The fact that the shifts in the absorbance and emission spectra follow the same binding curve as determine from mechanical manipulation in the MT strongly suggests that intercalation is the relevant binding mode for fluorescence, which is fully consistent with the molecular mechanism proposed for the fluorescence enhancement of unsymmetric cyanine dyes by impeding twisting about the monomethine bridge (57).

SYBR Gold is structurally related to SYBR Green I (Figure 1B) and thus to the family of unsymmetric cyanine dyes based on the thiazole orange (TO) chromophore. TO is composed of a benzothiazole and quinoline heterocycle coupled via a monomethine bridge (Figure 1A). Compared to TO, SYBR Gold has additional residues at position R1 and R3 and a benzoxazole moiety instead of a benzothiazole (Figure 1A) (57). The atom replacement in the core and varying substituents on the quinoline heterocycles impact the observed spectroscopic features and thus account for differences observed in comparison to other members of the family. It is, however, evident that the fluorogenic character of the dyes by nucleic acid intercalation is based on a similar photophysical mechanism (57–59). This mechanism is well described for TO (and its derivatives) where the fluorescence quantum yield is controlled by the competition of two pathways for excited state deactiviation: (i) non-radiative internal conversion via cis/trans isomerization around the central double bond and (ii) the radiative fluorescence transition. In aqueous solution, TO and all related derivatives are virtually non-fluorescent (59) since photoisomerization (i) is fully dominating the excited state deactivation. In more viscous solution, e.g. in glycerol, or upon interaction with nucleobases, photoisomerization, which requires a torsional motion around the monomethine bridge, becomes less efficient, giving rise to stronger fluorescence and longer excited state lifetimes.

The spectroscopic parameters we determined here for SYBR Gold are consistent with those of TO-based cyanine dyes including TO, Pico Green, and SYBR Green I (2). In general DNA complexation of the dyes to nucleic results in: (i) increased wavelength of the absorbance maximum, (ii) decreased wavelength of the emission maximum and overall narrower absorbance/emission bands, (iii) up to 1000-fold increase in fluorescence quantum yield and (iv) strongly increased excited state lifetimes (see below).

A clear difference between SYBR Gold and the other dyes of the same family is the much larger Stokes shift (∼50 nm for SYBR Gold versus ∼20 nm for the other dyes). The larger Stokes shift for SYBR Gold might in part be explained by the methoxy-substituent at position R3 (Figure 1A), by energetic stabilization of the fluorescent excited state via resonance effects. Similar findings were reported for (hemi)thioindigo photoswitches, where both the energetic position of the excited state minimum and its lifetime scaled with chemical substitutions at either sides of the chromophore (60–62). Alternatively, the formation of twisted-internal charge transfer states (TICT) (63,64) might account for variations of the Stokes shift, e.g. via varying R1 and R2 residues in SYBR Gold and SYBR Green I. Also the rotational freedom of the side chains R1–R3 in SYBR Gold and SYBR Green I in the electronic ground-state might have contributed towards spectral broadening of the free dye, which becomes restricted upon DNA intercalation (2). We hypothesize that the exchange of the benzothiazole moiety in SYBR Green I to benzoxazole in SYBR Gold has a relative minor impact on the fluorogenic character of the two dyes. This idea is supported by spectroscopic studies of indigoid photoswitches, where an exchange of nitrogen to sulfur in the hemiindigo-moeity preserved the overall photophysical and photochemical character of the photoswitches (30,65).

### Fluorescence intensity scales linearly with SYBR Gold intercalation at concentrations $ \le {\rm{\ }}2.5{\rm{\ \mu M}}$

**Figure 5.**
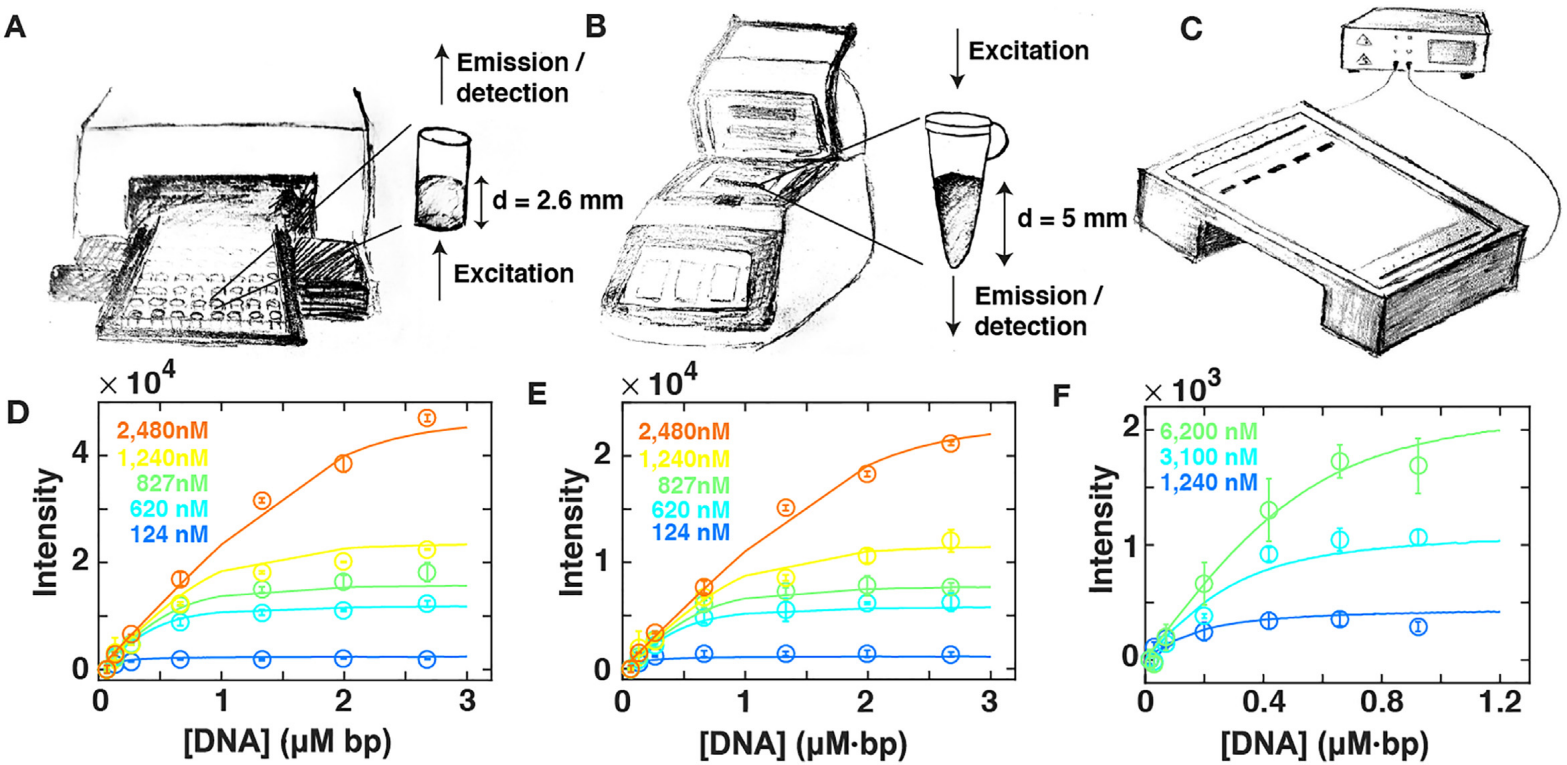
Fluorescence as a function of DNA and SYBR Gold concentrations below 2.5 μM dye. (**A**) Schematic of the 96-well plate reader setup: the SYBR Gold-DNA solution is inserted into the wells of the plate. The sample is excited from the bottom and the fluorescence intensity is measured from the top. The pathlength of the setup is *d* = (2.6 ± 0.05) mm. (**B**) Schematic of the qPCR cycler setup: the SYBR Gold-DNA solution is inserted into PCR tubes that are then placed in the thermal cycler. The sample is excited from the top and the fluorescence intensity is measured from the bottom. The pathlength of the setup is d = (5.0 ± 0.05) mm. (**C**) Schematic of the gel electrophoresis setup; the gel is stained with SYBR Gold after running. (**D**) Fluorescence intensities recorded using a plate reader and torsionally unstrained DNA (pBR322). The circles and error bars are the mean and standard deviation from at least two independent measurements. The solid line is the best fit of the finite concentration McGhee-von Hippel model (see Materials and Methods and Table 1). The SYBR Gold concentrations are (from blue to orange) 124, 620, 827, 1240, 2480 nM. (**E**) Fluorescence intensities recorded using a qPCR cycler. Same conditions and same fitting as for the plate reader data shown in (D). (**F**) Fluorescence intensities recorded using gel electrophoresis. Same fitting as for the plate reader data shown in (D). The SYBR Gold concentrations indicated in the legend text are of the staining solution. The actual concentrations in the gel are lower, by approximately 10-fold, as determined from fitting a global dilution factor (see Materials and Methods).

To further correlate our findings from DNA micromanipulation with SYBR Gold fluorescence and to determine guidelines for optimal quantification of DNA by SYBR Gold staining, we performed a series of experiments probing the interaction of SYBR Gold with DNA by monitoring bulk fluorescence intensity values. We measured fluorescence intensities as a function of DNA and SYBR Gold concentration using three different techniques: a plate reader, a qPCR cycler with fluorescence intensity readout, and gel electrophoresis (Methods, Figure 5A–C, and Supplementary Figure S7). The absorbance spectra showed that for concentrations ≤2.5 μM SYBR Gold, the absorbance is ≤0.1 (Supplementary Figure S3), such that inner filter effects (i.e. absorption of the excitation intensity and/or absorption of emitted photons before detection in the measurement cell) could be neglected (42) for our measurement geometries. Fluorescence intensities recorded for a range of DNA and SYBR Gold concentrations ≤2.5 μM increase with increasing DNA concentration for fixed dye concentration (data sets with different color codes in Figure 5D–F) and conversely also increased with increasing dye for fixed DNA concentration. The results obtained from the plate reader, the qPCR cycler, and gel electrophoresis are very similar (compare Figure 5D–F). The fluorescence data from the three measurement modalities were well described by the finite concentration McGhee-von Hippel model, Equation (8), with *K*_d_, $n$ and $\alpha$ as fitting parameters. We find good agreement between the fitted binding parameters for the three different fluorescence measurement modalities and also between the values obtained from single-molecule MT measurements of DNA mechanics and from the fluorescence intensities (Table 1). The close agreement between the binding parameters from mechanical manipulation and from fluorescence again strongly suggests that intercalation is the only binding mode of SYBR Gold to DNA that contributes to the fluorescence signal. In addition, the data suggest that the fluorescence intensity is linear in the number of intercalated SYBR Gold molecules, over a broad range of DNA dye molecules per DNA base pair, which in turn rules out significant effects on fluorescence from proximity of SYBR Gold molecules in the helix, e.g. through static quenching effects.

**Table 1.** McGhee-von Hippel model binding parameters determined from different data sets

	Dissociation constant *K*_d_ (μM)	Binding site size *n*	Fluorescence intensity scale *α*
Magnetic tweezers force-extension data	0.273 ± 0.026	1.67 ± 0.04	N.A.
Plate reader fluorescence	0.139	1.46	4.28 × 10^10^
qPCR cycler fluorescence	0.155	1.45	2.21 × 10^10^
Gel electrophoresis	0.191	1.57	7.59 × 10^9^

### Dynamic self-quenching limits the linear fluorescence response at high SYBR Gold concentrations

From bulk fluorescence intensity measurements at SYBR Gold concentrations >2.5 μM, fluorescence increases with increasing SYBR Gold concentration were lower than expected (Supplementary Figure S8). In a first step we used the available absorbance data (Supplementary Figure S3) to correct for the inner filter effect using the formula (42)

(10) $$\begin{equation*}{I_{\textrm{corr}}} = {I_{\textrm{obs}}} \cdot {10^{\left( {{A_{ex}} + {A_{em}}} \right)/2}}\end{equation*}$$

where ${I_{corr}}$ and ${I_{obs}}$ are the corrected and observed fluorescence intensity and ${A_{ex}}$ and ${A_{em}}$ the path-length (indicated in Figure 5) corrected absorbance values. Correcting for the inner filter effect increases the fluorescence values significantly in this concentration regime (Supplementary Figure S8, red circles). Nonetheless, even the corrected fluorescence values are still below the intensities observed at lower dye concentration and much below the values predicted by the finite concentration McGhee-von Hippel model using the parameters determined from the fits to the lower concentration data (Supplementary Figure S8, solid lines). This suggests that some form of self-quenching occurs. The observed quenching above 2.5 μM SYBR Gold is unlikely due to dye-dye interactions intercalated in the DNA helix, which have been described previously for other dyes (66), as the quenching is similar for different DNA concentration, which correspond to different loading densities in the DNA helix at a given dye concentration. In particular, we do not observe significant quenching at SYBR Gold concentrations <2.5 μM, even under conditions with a very high packing density of dye in the DNA helix (Figure 5D, E).

### Fluorescence lifetime measurements reveal dynamic quenching at high SYBR Gold concentrations

**Figure 6.**
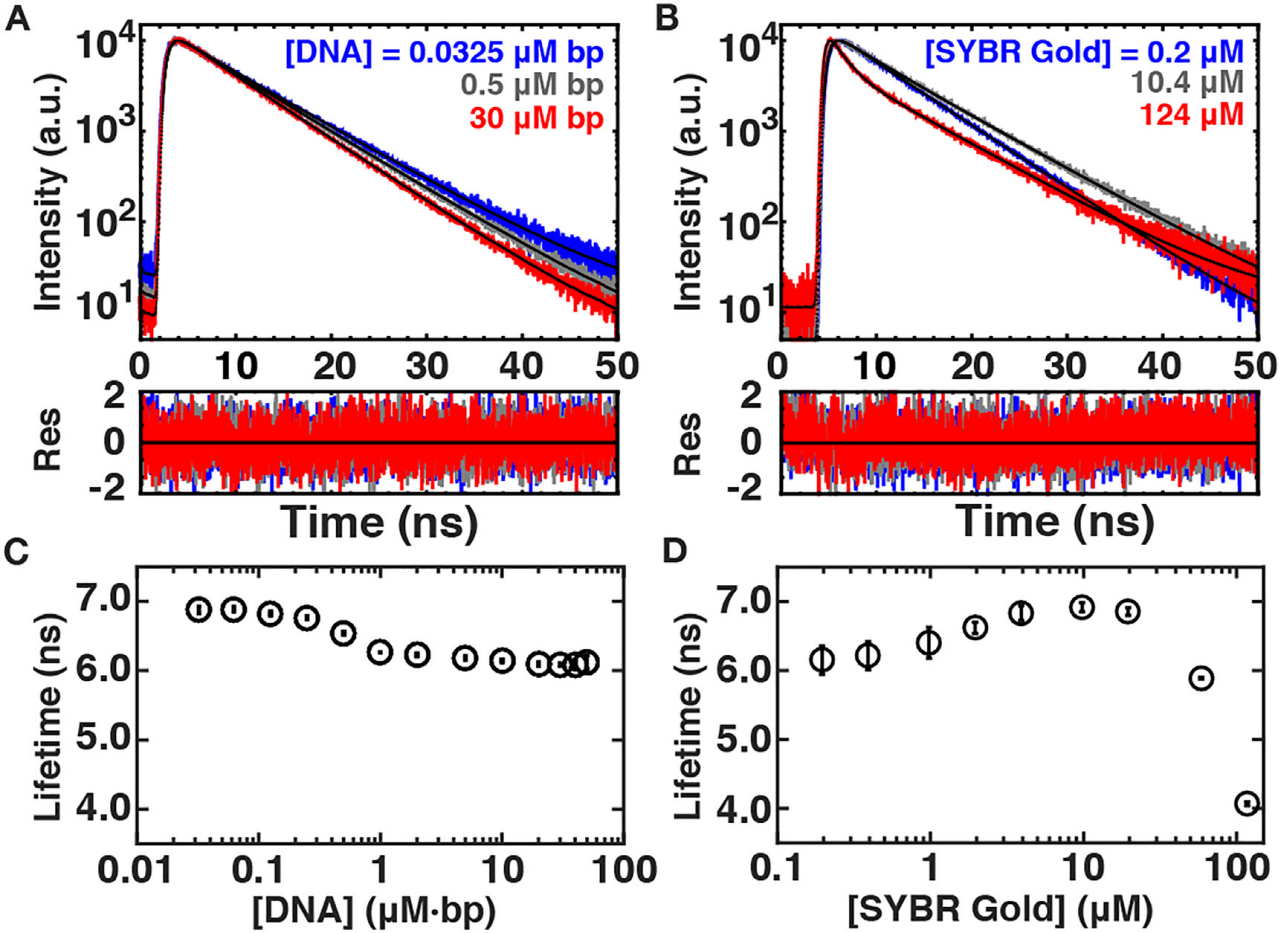
Fluorescence lifetime measurements. (**A**) Fluorescence lifetime measurements for 0.0325 (blue), 0.5 (grey) and 30 (red) μM⋅bp DNA at a SYBR Gold concentration of 2.5 μM. The data are fit by a single exponential decay. (**B**) Fluorescence lifetime measurements for 0.2 μM (blue), 10.4 μM (gray) and 124 μM (red) SYBR Gold in the presence of a constant DNA concentration of 2 μM⋅bp DNA. At high dye concentrations, the data show a clearly bi-exponential decay, therefore, we fit the data by a two-exponential model (Materials and Methods). (**C**) Fluorescence lifetimes as a function of DNA concentration determined from single-exponential fits as in (A). (**D**) Amplitude weighted fluorescence lifetime as a function of SYBR Gold concentration from measurements at 2 μM⋅bp DNA determined from two exponential fits (Materials and Methods).

To better understand the mechanism of self-quenching, we determined fluorescence lifetimes of SYBR Gold by time-correlated single photon counting. In a first set of experiments we varied the DNA concentration at a constant SYBR Gold concentration of 2.5 μM. In the absence of DNA, SYBR Gold shows a time-correlated single photon counting histogram that cannot be distinguished from the instrumental response function of our system. DNA binding results in a single exponential fluorescence decay (Figure 6A, C) with a lifetime in range of ∼6.5 ns. This lifetime value is similar, but slightly larger than the values reported previously (2). The difference might be due to variations in data analysis (tail fitting vs. reconvolution fitting) and experimental conditions (DNA sample and buffer). At constant SYBR Gold concentration of 2.5 μM, lifetimes vary from ∼6.9 ns at high loading ratios >1:2 to ∼6.1 ns at lower loading density of dye in the DNA helix (<1:10). This is consistent with the absence of dynamic quenching between SYBR Gold molecules along the DNA helix, since this would likely lead to a reduction in the lifetime with increasing packing density (*i.e*. lower DNA concentration), while we experimentally observe an increase (Figure 6C). The slightly longer lifetime at high loading densities is consistent with a stabilization of the excited state and with the observed red shift in the emission (Figure 4B), possibly caused by the stiffening of the helix at high packing densities (Figure 2D).

In a second set of measurements we varied the SYBR Gold concentration while keeping the DNA concentration constant at 2 μM⋅bp (Figure 6B, D). For SYBR Gold concentrations ≤10 μM, fluorescence decays are well described by single exponential fits, and the fitted lifetimes slightly increase up to ∼6.9 ns with increasing SYBR Gold concentration (Figure 6B). However, at SYBR Gold concentrations >10 μM, the lifetimes are no longer well-described by a single exponential fit. Instead, we see an additional faster decaying component appearing (Figure 6B). For consistency, we fitted the entire data set with a two-component model, with two exponential decays (described in detail in the Materials and Methods section). For simplicity, we report an amplitude weighted overall decay constant in Figure 6D and the individual decay times and amplitudes in Supplementary Figure S9.

The observation of a reduction in lifetime, together with a reduction in fluorescence intensity (Supplementary Figure S8), for SYBR Gold concentrations >2.5 μM at constant DNA concentration suggests a dynamic self-quenching mechanism. Since the data disfavor a self-quenching by intercalated dyes in the DNA helix, a more likely scenario is a dynamic quenching mechanism involving non-intercalated SYBR Gold. A quenching mechanism from free SYBR Gold in solution would imply for our data at the highest SYBR Gold concentration with a quenching decay time constant of ∼1 ns (Supplementary Figure S9) and a free dye concentrations of ∼100 μM an apparent bimolecular quenching constant of (∼1 ns ⋅ 100 μM)^−1^ ∼10^13^ M^−1^ s^−1^, which is roughly three orders of magnitude larger than diffusion controlled on-rates (42). The most likely explanation of dynamic quenching of the intercalated SYBR Gold is that quenching occurs from SYBR Gold molecules kept in close proximity to the DNA helix, possibly due to electrostatic interactions, favoured by the fact that SYBR Gold carries two positive charges (Figure 1A) and DNA is highly negatively charged (67). Charge interactions or, possibly, a combination of charge interactions and other association modes of SYBR Gold with the DNA helix will increase the local effective concentration of SYBR Gold around DNA and could, therefore, facilitate dynamic quenching (42).

### Recommendations for quantitation of DNA using SYBR Gold staining

**Figure 7.**
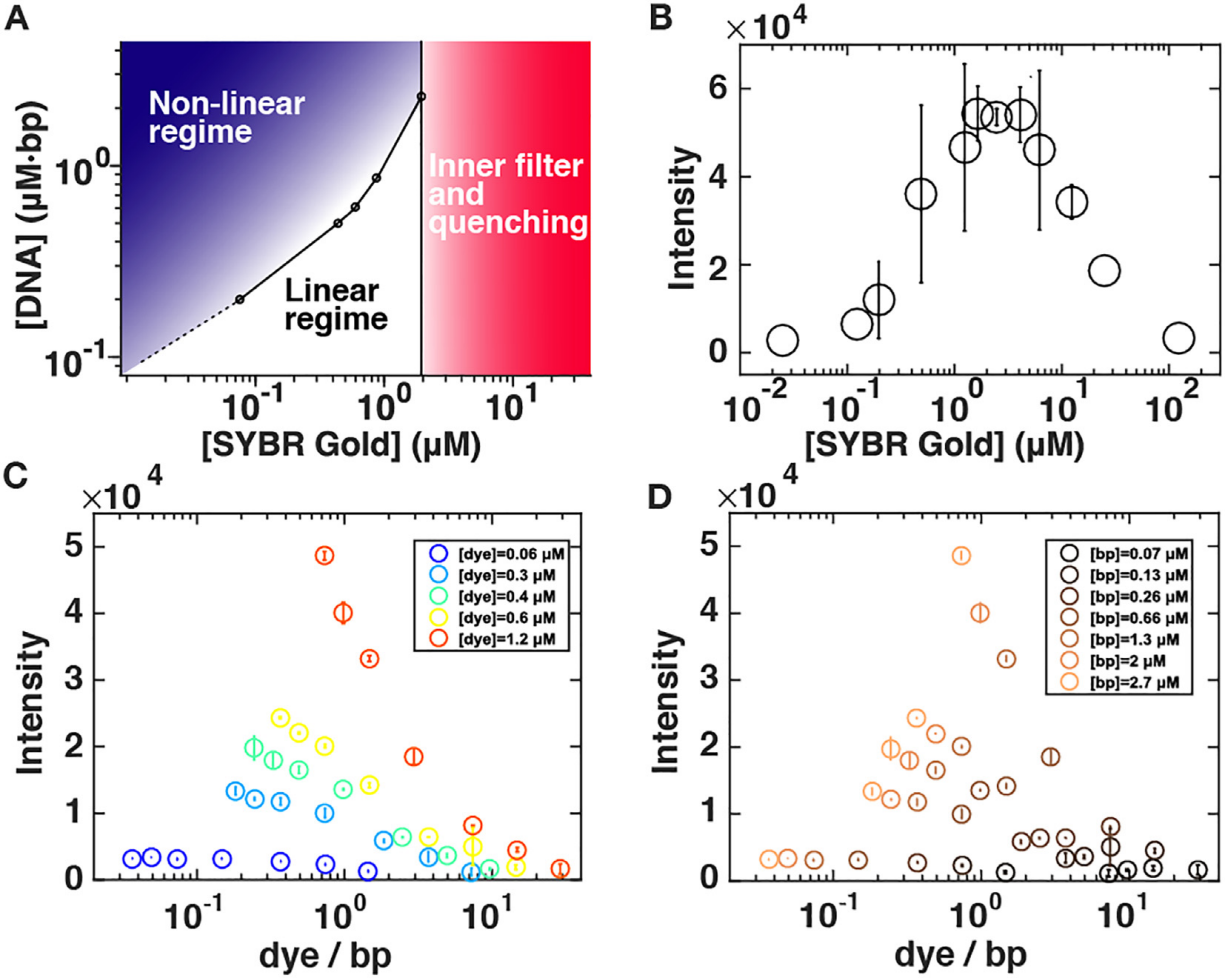
Recommendations for DNA quantitation by SYBR Gold fluorescence. (**A**) Phase diagram depicting different regimes of DNA detection by SYBR Gold fluorescence, as a function of SYBR Gold and DNA concentrations. (**B**) Fluorescence intensity recorded using a qPCR cycler at constant DNA concentration (Lambda DNA, 2.7 μM bp) at varying SYBR Gold concentrations. The data points are the mean values of two independent experiments including their standard deviations or stem from just one experiment. (**C**) Fluorescence intensity recorded using a plate reader as a function of the dye per base ratio. The circles and error bars are the mean and standard deviation from at least two independent measurements. The SYBR Gold concentrations are (from blue to orange) 0.124, 0.62, 0.83, 1.24, 2.48 μM. These are the same data as in Figure 5D plotted as a function of dyes per bp. (**D**) Fluorescence intensity recorded using a plate reader as a function of the dye per base ratio. The circles and error bars are the mean and standard deviation from at least two independent measurements. The DNA base pair concentrations are (from dark to light brown) 0.07, 0.13, 0.26, 0.66, 1.3, 2, 2.7 μM.

Our single-molecule MT and fluorescence spectroscopy assays provide a comprehensive view of DNA-SYBR Gold interactions. This knowledge enables us to provide practical guidelines for optimal DNA detection and quantitative DNA sensing applications using SYBR Gold. For quantitative assays, it is desirable to have a linear relationship between fluorescence intensity and DNA concentration. The optimal SYBR Gold concentration to ensure a linear relation between DNA concentration (up to DNA concentrations of ≈ 2 μM⋅bp or 1.3 ng/μl) and fluorescence intensity as well as an optimal sensitivity for DNA detection is at 2.5 μM (≈1:5000 dilution of the stock solution). This value for the optimal SYBR Gold concentration is 2× larger than the manufacturer's recommendation of 1:10 000-fold dilution. Reliable detection is possible with lower dye concentrations, however, with a reduced range for a linear fluorescence-DNA concentration response (Figure 7A). So if linearity and high signal are important, using high dye concentrations is desirable. However, at very high dye concentration (SYBR Gold concentrations > 2.5 μM) quenching and inner filter effects become relevant for typical measurement setups and need to be corrected for to get accurate and quantitative results (Figure 7A,B). For many measurements, it is likely beneficial to avoid inner filter and quenching effects by keeping the SYBR Gold concentration ≤2.5 μM.

In addition, we note that that the fluorescence intensity depends, in general, on both the dye and DNA concentration. Reporting concentrations as ‘dye per base pair’—as is frequently done for DNA stains—is problematic, since a large range of intensities correspond to the same dye per base pair ratio (Figure 7C, D) and since the connection between dye to base pair ratio and fluorescence intensity is many-to-one. Therefore, we advise to report both DNA and dye concentrations explicitly.

## DISCUSSION

We have solved the structure of SYBR Gold by NMR spectroscopy and mass spectrometry and employed single-molecule MT that provide rotational control in addition to control of the stretching forces to probe the binding properties of SYBR Gold to DNA. The single-molecule MT assay reveals systematic lengthening (up to 1.7 times the DNA contour length in the absence of dye) and unwinding of DNA (19.1° ± 0.7° per SYBR Gold bound) upon SYBR Gold binding. The mechanical signature strongly suggests intercalation, with an unwinding angle at the low end of the range of previously investigated intercalators. Fitting the McGhee–von Hippel model to the MT data, we find a binding constant *K*_d_ = (2.73 ± 0.26) × 10^–7^ M and binding site size of $n$ = 1.67 ± 0.04. These findings are in good agreement with the parameters from fluorescence intensity experiments for dye concentrations of up to ∼2.5 μM suggesting that the intercalative binding mode is responsible for the observed fluorescence. Additionally, we find that for SYBR Gold concentrations >2.5 μM, fluorescence quenching and inner filter effects become relevant. While we see no evidence for quenching between dyes intercalated in the helix, our data provide clear evidence for dynamic quenching from dyes in a loosely bound (likely at least partially driven by electrostatics) “cloud” around the DNA. Overall, we found that while SYBR Gold is advantageous due to its high quantum yield and brightness, it has a relatively narrow range of concentrations that strike a balance between avoiding inner filter effects and quenching, while staining DNA with a linear fluorescence to DNA concentration relationship. In summary, our work shows how using complementary techniques can provide a highly quantitative and comprehensive view of DNA-small molecule interactions and we anticipate our approach to be broadly applicable to other DNA binding agents.

## DATA AVAILABILITY

Data are available upon request.

## Supplementary Material

gkab265_Supplemental_File
