# Correction to ‘Molecular structure, DNA binding mode, photophysical properties and recommendations for use of SYBR Gold’

**DOI:** 10.1093/nar/gkab1164

**Published:** 2021-11-24

**Authors:** Pauline J Kolbeck, Willem Vanderlinden, Gerd Gemmecker, Christian Gebhardt, Martin Lehmann, Aidin Lak, Thomas Nicolaus, Thorben Cordes, Jan Lipfert

**Affiliations:** Department of Physics and Center for NanoScience, LMU Munich, Amalienstrasse 54, 80799 Munich, Germany; Department of Physics and Center for NanoScience, LMU Munich, Amalienstrasse 54, 80799 Munich, Germany; Bavarian NMR Center (BNMRZ), Department of Chemistry, Technical University of Munich, Garching, Germany; Physical and Synthetic Biology, Faculty of Biology, LMU Munich, Planegg-Martinsried, Germany; Plant Molecular Biology, Faculty of Biology, LMU Munich, Planegg-Martinsried, Germany; Department of Physics and Center for NanoScience, LMU Munich, Amalienstrasse 54, 80799 Munich, Germany; Department of Physics and Center for NanoScience, LMU Munich, Amalienstrasse 54, 80799 Munich, Germany; Physical and Synthetic Biology, Faculty of Biology, LMU Munich, Planegg-Martinsried, Germany; Department of Physics and Center for NanoScience, LMU Munich, Amalienstrasse 54, 80799 Munich, Germany

The published version of our article (1) contains two errors that we would like to correct. The full name of the compound SYBR Gold was incorrectly stated in the Abstract (page 5143) and in the Results section (on page 5148). The correct name is 2-(4-{[diethyl(methyl)ammonio]methyl}phenyl)-6-methoxy-1-methyl-4-{[(2Z)-3-methyl-1,3-benzoxazol-2-ylidene]methyl}quinolin-1-ium

In addition, structures that were shown in Figure 1A and B are drawn incorrectly. A corrected version of Figure 1 is enclosed below, the figure legend is not changed. Neither of these errors affects the conclusions in the paper, as all analyses were based on the correct structures.

**Figure 1.**
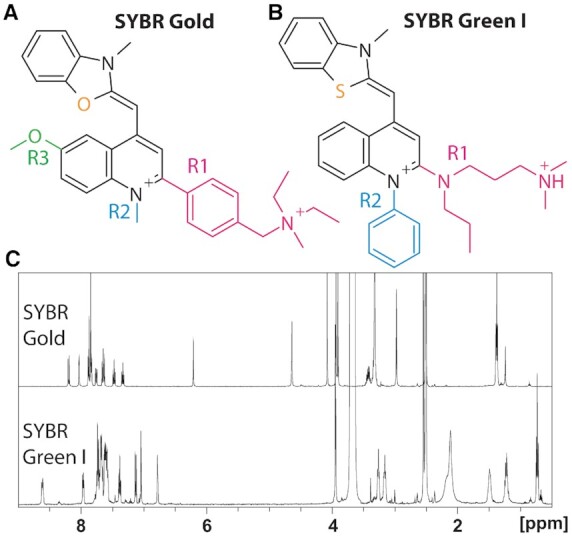
SYBR Gold structure and ^1^H NMR spectra. (**A**) The structure of SYBR Gold as determined by NMR studies and mass spectrometry. For clarity, the side chains are shown in color and named R1, R2, R3. (**B**) The structure of SYBR Green I from (29). The protonation state of the side chain R1 is for aqueous solution near neutral pH. (**C**) ^1^H NMR spectra of SYBR Gold and SYBR Green I recorded in DMSO-d6.

We would like to thank the reader who brought the errors to our attention and apologize for any confusion that they might have caused.

## References

[1] Kolbeck P.J. , Vanderlinden W., Gemmecker G., Gebhardt C., Lehmann M., Lak A., Nicolaus T., Cordes T., Lipfert J. Molecular structure, DNA binding mode, photophysical properties and recommendations for use of SYBR Gold. Nucleic Acids Res. 2021; 49:5143–5158. 33905507 10.1093/nar/gkab265 PMC8136779

[29] Zipper H. , Brunner H., Bernhagen J., Vitzthum F. Investigations on DNA intercalation and surface binding by SYBR Green I, its structure determination and methodological implications. Nucleic Acids Res. 2004; 32:e103. 15249599 10.1093/nar/gnh101 PMC484200

