# Supplementary material for: Molecular structure, DNA binding mode, photophysical properties and recommendations for use of SYBR Gold
Source: Nucleic Acids Res. 2021 Apr 27;49(9):5143–58. doi: 10.1093/nar/gkab265 (PMC8136779; doi:10.1093/nar/gkab265)
Supplement: gkab265_Supplemental_File [file gkab265_supplemental_file.pdf]

# SUPPLEMENTARY INFORMATION FOR

# Molecular structure, DNA binding mode, photophysical properties, and recommendations for use of SYBR Gold

Pauline J. Kolbeck[1], Willem Vanderlinden[1,*], Gerd Gemmecker[2], Christian Gebhardt[3], Martin Lehmann[4], Aidin Lak[1], Thomas Nicolaus[1], Thorben Cordes[3], and Jan Lipfert[1,*]

[1]Department of Physics and Center for NanoScience, LMU Munich, Amalienstrasse 54, 80799 Munich, Germany.

[2]Bavarian NMR Center (BNMRZ), Department of Chemistry, Technical University of Munich, Garching, Germany

[3]Physical and Synthetic Biology, Faculty of Biology, LMU Munich, Planegg-Martinsried, Germany

[4]Plant Molecular Biology, Faculty of Biology, LMU Munich, Planegg-Martinsried, Germany

*To whom correspondence should be addressed:

Willem Vanderlinden: Email: Willem.Vanderlinden@physik.uni-muenchen.de; Phone: +49-89-2180-3545

Jan Lipfert: Email: Jan.Lipfert@lmu.de; Phone: +49-89-2180-2005

**Supplementary Table S1.** Absorbance values for different SYBR Gold stocks

| Stock | LOT number | Extrapolated absorbance at 495 nm measured via UV-Vis |
|---|---|---|
| 1 | 2068280 | 496 ± 5 |
| 2 | 2098432 | 431 ± 6 |
| 3 | 2174893 | 640 ± 8 |
| 4 | 2174893 | 703 ± 10 |

**Supplementary Table S2.** NMR shifts assignment table for SYBR dyes.

| | SYBR Safe* | Thiazole Orange* | SYBR Green-I | SYBR Green-II | SYBR Gold (DMSO) | SYBR Gold (CD3OD) | SYBR Gold simulation |
|---|---|---|---|---|---|---|---|
| **Quinoline** | | | | | | | |
| **1-CH2(H)α** | 4.57 | | | | | | |
| | *55.40* | | | | | | |
| **1-CH2β** | 1.89 | | | | | | |
| | *22.10* | | | | | | |
| **1-CH3γ** | 0.96 | | | | | | |
| | *10.60* | | | | | | |
| **N1** | | | 153.0 | 160.3 | 149.0 | 122.0 | |
| **N1-CH3** | | 4.17 | | | 3.94 | 4.02 | 3.95 |
| | | *42.30* | | | *40.40* | *39.00* | *37.30* |
| **2** | 8.64 | 8.61 | | | | | |
| | *144.40* | *145.00* | *158.50* | *158.10* | *152.00* | *151.70* | *152.00* |
| **3** | 7.39 | 7.36 | 7.04 | 8.00 | 7.85 | 8.04 | 7.98 |
| | *107.70* | *107.70* | *102.80* | *106.20* | *112.00* | *112.10* | *119.20* |
| **4** | *148.50* | *148.50* | *149.10* | *148.40* | *148.50* | *149.20* | *130.80* |
| **4a** | | | | | | | |
| | *124.20* | *123.90* | *122.00* | *121.70* | *124.90* | *125.00* | *126.00* |
| **5** | 8.81 | 8.80 | 8.61 | 8.76 | 8.02 | 7.94 | 7.54 |
| | *125.70* | *125.40* | *125.50* | *126.10* | *106.80* | *105.30* | *106.40* |
| **6** | 7.76 | 7.78 | 7.58 | 7.65 | | | |
| | *126.70* | *126.80* | *126.10* | *126.40* | *158.30* | *158.50* | *156.50* |
| **6-OCH3** | | | | | 4.08 | 4.12 | 4.00 |
| | | | | | *56.80* | *55.30* | *55.50* |
| **7** | 7.99 | 8.02 | 7.67 | 7.74 | 7.75 | 7.72 | 6.93 |
| | *133.20* | *133.10* | *133.00* | *133.60* | *123.60* | *123.60* | *123.80* |
| **8** | 8.17 | 8.05 | 7.13 | 6.83 | 8.19 | 8.14 | 7.34 |
| | *118.10* | *118.20* | *119.00* | *118.70* | *121.40* | *120.00* | *128.70* |
| **8a** | *137.00* | *137.90* | *140.70* | *141.20* | *134.80* | *134.70* | *143.50* |
| **Benzo-X-azole** | X=S | X=S | X=S | X=O | X=O | X=O | X=O |
| **2′** | *160.00* | *159.70* | *158.90* | *162.10* | *161.80* | *161.90* | *161.40* |
| **2a′** | 6.94 | 6.93 | 6.78 | 6.33 | 6.21 | 6.17 | 8.06 |
| | *88.00* | *87.80* | *87.20* | *73.90* | *74.10* | *73.10* | *113.70* |
| **N3'** | | | 140.0 | 127.9 | 126.5 | 112.7 | |
| **3′-CH3** | 4.03 | 4.01 | 3.95 | 3.93 | 3.91 | 3.90 | 3.93 |
| | *33.70* | *33.70* | *33.90* | *31.10* | *31.10* | *29.40* | *37.80* |
| **3a′** | *140.40* | *140.40* | *141.20* | *131.80* | *131.90* | *131.40* | *132.60* |
| **4′** | 7.80 | 7.77 | 7.70 | 7.69 | 7.63 | 7.53 | 7.82 |
| | *112.90* | *112.80* | *112.90* | *111.30* | *111.10* | *109.80* | *110.90* |
| **5′** | 7.63 | 7.61 | 7.59 | 7.52 | 7.47 | 7.48 | 7.13 |
| | *128.10* | *128.00* | *128.70* | *126.50* | *126.40* | *125.90* | *126.10* |
| **6′** | 7.43 | 7.41 | 7.39 | 7.44 | 7.33 | 7.35 | 7.10 |
| | *124.40* | *124.30* | *124.60* | *124.90* | *124.50* | *124.00* | *122.50* |
| **7'** | 8.06 | 8.04 | 7.97 | 7.80 | 7.65 | 7.59 | 6.89 |
| | *122.80* | *122.80* | *123.20* | *111.40* | *111.20* | *110.30* | *110.40* |
| **7a'** | *123.80* | *123.70* | *123.80* | *146.50* | *146.40* | *146.50* | *147.60* |

| | SYBR Safe* | Thiazole Orange* | SYBR Green-I | SYBR Green-II | SYBR Gold (DMSO) | SYBR Gold (CD3OD) | SYBR Gold simulation |
|---|---|---|---|---|---|---|---|
| **Tail (R1)** | | | | | | | |
| **α** | | | | | | | |
| | | | ***N:*** *84.2* | | *137.10* | *137.50* | *136.70* |
| **β / β'** | | | 3.26 / 3.16 | 3.49 | 7.88 | 7.89 | 7.89 |
| | | | *50.4 / 54.8* | *31.8* | *130.10* | *129.80* | *128.50* |
| **γ / γ'** | | | 1.49 / 1.23 | 2.71 | 7.84 | 7.91 | 7.36 |
| | | | *24.7 / 20.3* | *56.8* | *134.10* | *133.60* | *127.80* |
| **δ / δ'** | | | *2.19 / 0.73* | | | | |
| | | | *56.3 / 11.5* | ***N:*** *26.2* | *130.50* | *129.85* | *130.50* |
| **Nδ-CH3** | | | | 2.20 | | | |
| | | | | *45.3* | | | |
| **ε** | | | | | 4.64 | 4.72 | 4.39 |
| | | | ***N:*** *23.7* | | *63.50* | *63.80* | *65.30* |
| **Nε-CH3** | | | 2.12 | | | | |
| | | | *45.2* | | | | |
| **Nζ** | | | | | 61.4 | 87.5 | |
| **Nζ-CH3** | | | | | 2.97 | 3.10 | 2.86 |
| | | | | | *46.70* | *46.10* | *48.20* |
| **η** | | | | | 3.33/3.43 | 3.49/3.56 | 3.15 |
| | | | | | *55.80* | *55.90* | *54.30* |
| **θ** | | | | | 1.38 | 1.53 | 1.34 |
| | | | | | *8.90* | *6.90* | *8.40* |

*taken from (1)

Boxed areas mark chemical shifts with large differences between benzo-thiazoles and benzo-oxazoles, with good agreement between measured and simulated data for SybrGold (for $^{15}N3$ no simulation is available; for position 2a' the simulation fails completely for both the $^{1}H$ and $^{13}C$ shifts). The naming of the atoms can be taken from Supplementary Figure S2.

**Supplementary Table S3.** McGhee-von Hippel model binding parameters for different SYBR dyes determined by single-molecule magnetic tweezers experiments.

| | Dissociation constant $K_d$ (μM) | Binding site size $n$ (bp) | Unwinding angle $\theta$ (°) |
|---|---|---|---|
| SYBR Gold* | 0.216 ± 0.021 | 1.67 ± 0.04 | 19.1 ± 0.7 |
| SYBR Green I* | 0.203 ± 0.037 | 1.77 ± 0.06 | 19.3 ± 1.3 |
| Pico Green† | 0.943 ± 0.050 | 2.32 ± 0.21 | 21 ± 14 |

* this work

†Ref. (2)

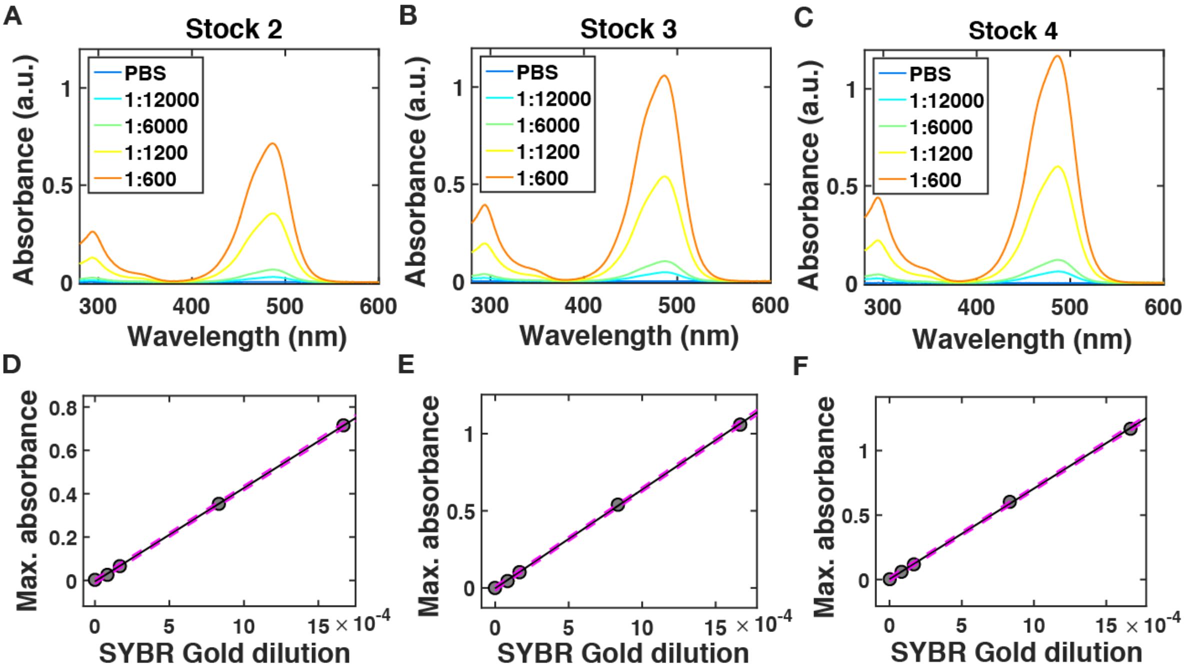


**Supplementary Figure S1. Absorbance of different SYBR Gold stocks. A-C)** Absorbance spectra for varying SYBR Gold dilutions (from blue to orange: PBS, 1:12000, 1:6000, 1:1200, 1:600) for three different SYBR Gold stocks purchased from Invritrogen (LOT numbers 2098432, 2174893, 2174893). Spectra shown are recorded in the absence of DNA. The maximum absorbance is at a wavelength of 486 nm. **D-F)** Value of the absorbance maxima of the SYBR Gold absorbance spectra as a function of the SYBR Gold concentration. The circles are the experimental data, the solid line is a linear fit; extrapolation of the linear fit yields the value for the undiluted SYBR Gold which is found to be 413 ± 6, 640 ± 8, 703 ± 10 (**Supplementary Table S1**).

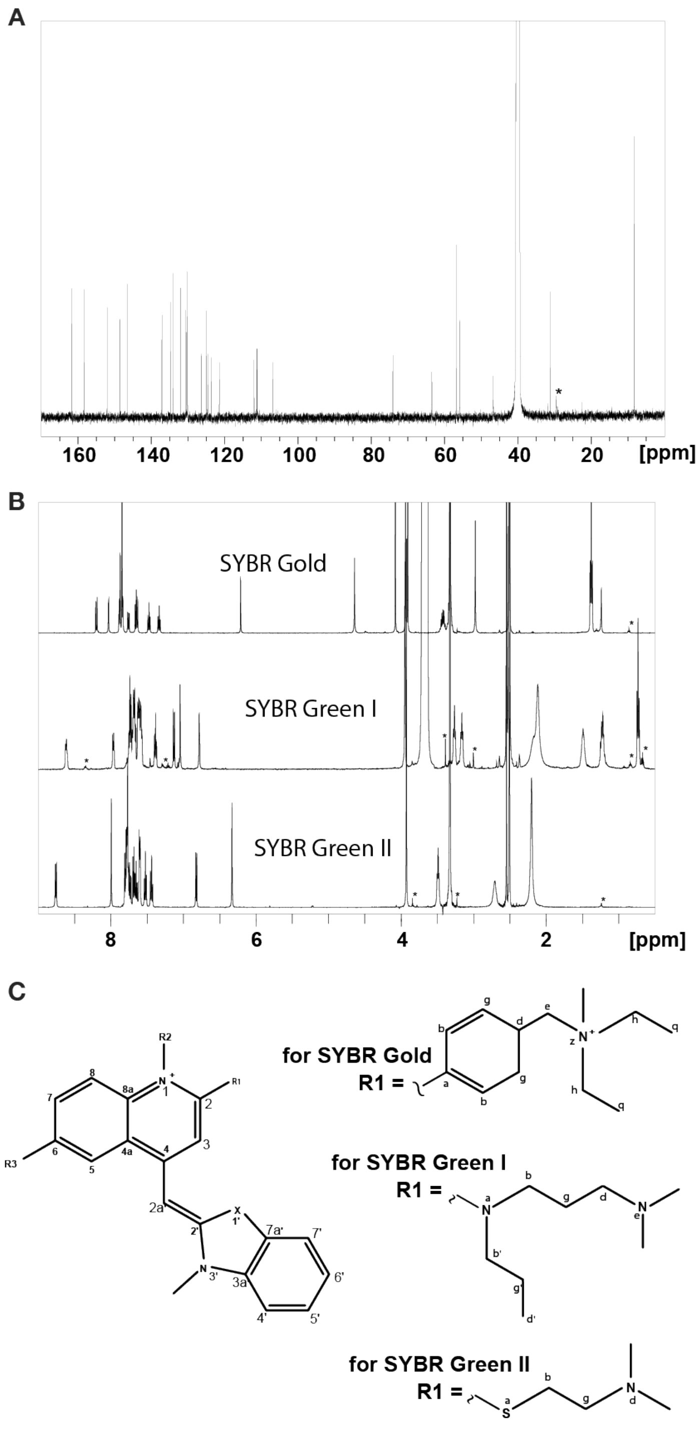


**Supplementary Figure S2. SYBR Gold NMR spectra. A)** 1D $^{13}C$ NMR spectrum of SYBR Gold in DMSO-d6, AV III 500 MHz (cryo). The peak highlighted with a star is a contamination. **B)** 1D $^{1}H$ spectra of SYBR Gold, SYBR Green I, and SYBR Green II in DMSO-d6 (298 K, MHz; SYBR Gold and SYBR Green II 1 scan, SYBR Green I 16 scans). Impurities identified by DOSY experiments are marked with an asterix *. **C)** The core structure of the SYBR family of dyes and the side chain R2 for SYBR Gold, SYBR Green I, and SYBR Green II. For clarity, we labelled all atoms and the side chains R1, R2, R3. The same nomenclature is used in the NMR shifts assignment table for SYBR dyes (**Supplementary Table S2**).

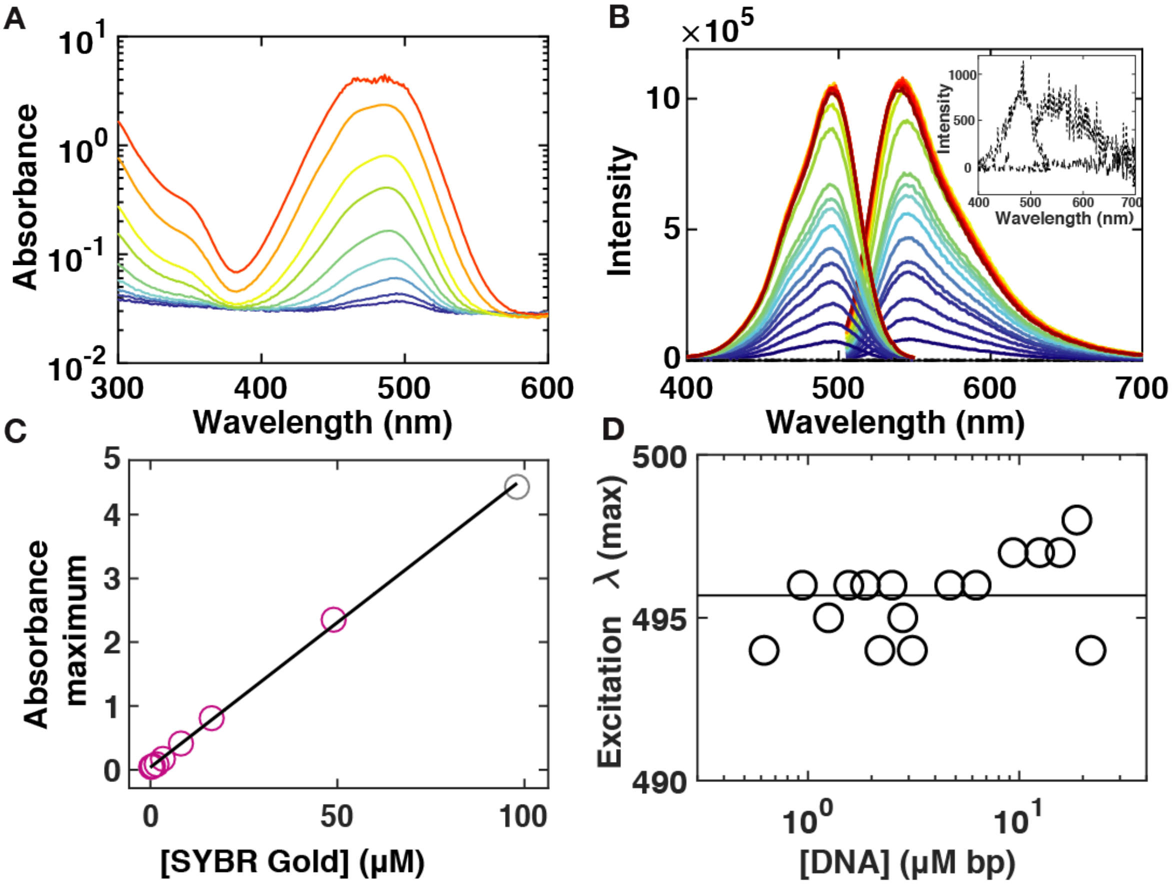


**Supplementary Figure S3. SYBR Gold absorbance, excitation, and emission spectra. A)** Absorbance spectra for varying SYBR Gold concentrations (from blue to red: 0.16, 0.33, 0.82, 1.6, 3.3, 8.2, 16, 49, 98 μM) in the presence of 2 μM·bp DNA (Lambda-DNA (NEB)). The peak of the spectrum at the highest concentration is noisy, as the dynamic range of the instrument is approached. Points from this spectrum are included below in panel C (as well as **Figure 4A** in the main text) but greyed out. **B)** SYBR Gold excitation and emission spectra at constant SYBR Gold concentration (2.5 μM) and varying DNA concentration (from blue to red: 0.9, 1.3, 1.6, 1.9, 2.2, 2.8, 3.1, 4.7, 6.3, 9.4, 12.5, 15.6, 18.8, 21.9, 25.0, 28.1, 31.3 μM·bp, dotted line: SYBR Gold only, dashed line: PBS only. The inset shows PBS only and SYBR Gold only spectra on a different scale for clarity. **C)** Value of the absorbance maxima of the SYBR Gold absorbance spectra as a function of the SYBR Gold concentration. The circles are the experimental data, the solid line is a linear fit (R = 0.9997); extrapolation of the linear fit yields the value for the undiluted SYBR Gold which is found to be 435 ± 6 and implies a concentration of the undiluted SYBR Gold of (9.8 ± 0.1) mM. This is significantly different from the stock concentration we determined for other SYBR Gold stocks (**Supplementary Figure S1**) **D)** Position of the excitation maxima at constant SYBR Gold concentration (2 μM) and varying DNA concentrations. No significant shift is observed for the excitation maxima. The line is simply the mean of the data points at all concentrations, which is 495.7 nm ± 1 .25 nm (mean ± std).

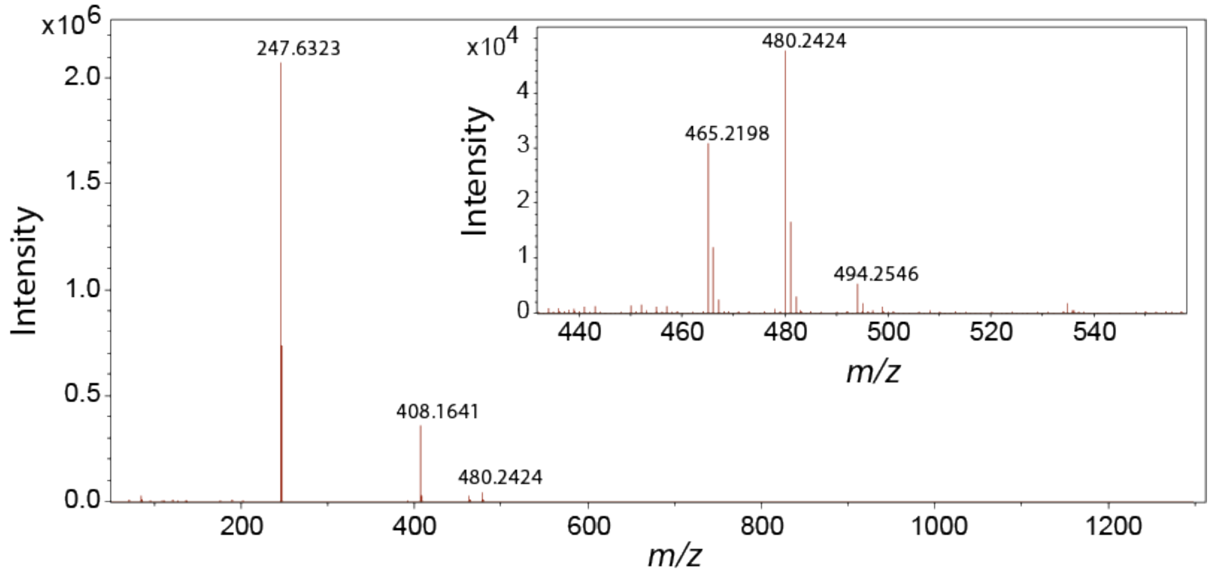


**Supplementary Figure S4. Mass spectrometry analysis of SYBR Gold.** The main population is at *m/z* = 247.6. Minor populations are found at *m/z* of 408.2 and around 480. The inset has a zoom into the region around *m/z* = 480.

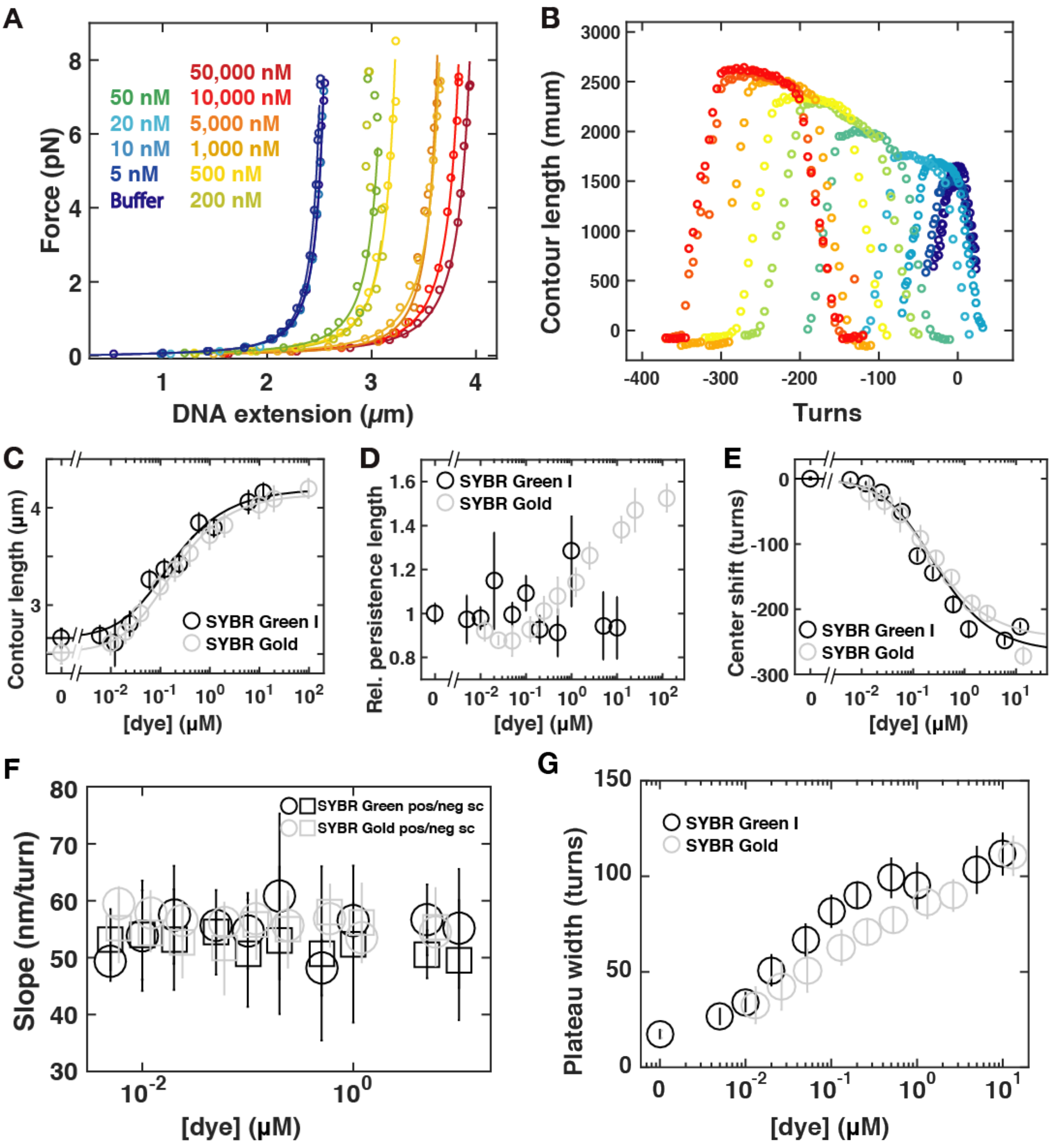


**Supplementary Figure S5. Effects of SYBR Green I on DNA force-extension behaviour, twist and torque.**

**A)** Force-extension curves for 7.9-kb DNA in the presence of increasing concentrations of SYBR Green (increasing concentrations from blue to red indicated in the figure legend). Symbols are raw data, lines are fits of the WLC model. A systematic increase the DNA extension with increasing SYBR Gold concentration is apparent. **B)** Rotation-extension curves for 7.9 kb DNA at $F$ = 0.5 pN in the presence of increasing concentrations of SYBR Green. The SYBR Green concentrations are (from blue to red) 0, 5, 10, 20, 50, 100, 200, 500, 1000, 5000 1000, 50000 nM. With increasing concentrations of SYBR Gold the rotation curves shift to negative turns; the DNA length at the center of the curves increases; and the rotation-extension curves broaden.

**C)** DNA contour length determined from fits of the WLC model as a function of SYBR Green concentration. The black line is a fit to the McGhee–von Hippel model (reduced $\chi^2$ =

0.75), with a dissociation constant $K_d$ = 0.20 ± 0.04 µM and a binding site size n = 1.77 ± 0.06. We show the corresponding data for SYBR Gold in grey as a comparison (**Fig 2C**). **D)** DNA bending persistence length from WLC fits measured as a function of the dye concentration, indicating that the persistence length stays constant with increasing dye concentration. We show the corresponding data for SYBR Gold in grey as a comparison (**Fig 2D**). **E)** Quantification of the shift in the center position of the rotation-extension curves as a function of the SYBR Green concentration. The center positions were determined from fitting slopes in the positive and negative plectonemic regime and by computing the intersection of the two slopes. The black line is a fit of the McGhee-von Hippel model (reduced $\chi^2$ = 5.5), with the dissociation constant $K_d$ and binding site size $n$ set to the values determined from the force extension data (**Panel A**) and the unwinding angle per SYBR Gold intercalation determined from the fit to be $\Delta\theta$ =19.3° ± 1.3. We show the corresponding data for SYBR Gold in grey as a comparison (**Fig 3B**). **F)** Extension vs. turn slopes in the plectonemic regime (determined as indicated by the red lines in **Supplementary Figure Fig S6A**) as a function of SYBR Green concentration for positive (circles) and negative (squares) plectonemic supercoils. We show the corresponding data for SYBR Gold in grey as a comparison (**Supplementary Figure Fig S6B**). **G)** Width of the pre-buckling regime (in turns) vs. SYBR Green concentration. In grey we show the corresponding data for SYBR Gold in grey as a comparison (**Supplementary Figure Fig S6C**). In panel A and B one typical experiment is shown for clarity. Data points and error bars for SYBR Green in the panels C to G are the mean and standard deviation from at least 24 independent measurements.

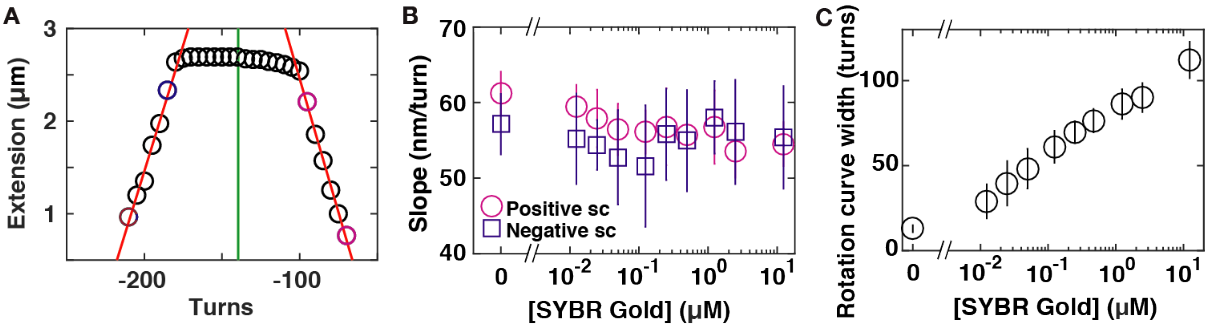


**Supplementary Figure S6. SYBR Gold rotation curve analysis. A)** Example of a rotation-extension curve and analysis of center positions and slopes. Data are for 7.9 kb DNA at $F$ = 0.5 pN in the presence of 496 nM SYBR Gold (circles). The center positions are determined from fitting slopes in the positive and negative plectonemic regime (red lines) and by computing the intersection of the two slopes (indicated by the green line). **B)** Extension vs. turn slopes in the plectonemic regime (determined as indicated by the red lines in panel A) as a function of SYBR Gold concentration for positive (red circles) and negative (blue squares) plectonemic supercoils. **C)** Width of the pre-buckling regime (in turns) vs. SYBR Gold concentration. Data points and error bars in panel B and C are the mean and standard deviation from at least 14 independent measurements. In panel A one typical experiment is shown for clarity.

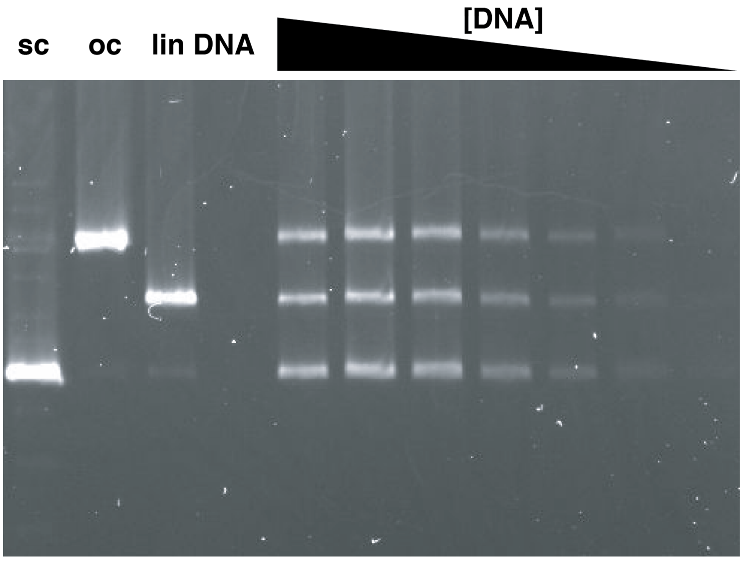


**Supplementary Figure S7. Gel electrophoresis of PBR322 plasmid DNA and visualization by SYBR Gold fluorescence.** Lanes from left to right: supercoiled DNA, open-circular DNA, linear DNA, empty lane, and equimolar mixtures of supercoiled, open-circular, and linear DNA with decreasing amounts of DNA: 30 ng, 21.4 ng, 13.6 ng, 6.5 ng, 2.3 ng, 1.0 ng, 0.5 ng. The gel was stained after electrophoretic separation with 3.1 μM SYBR Gold in TAE buffer (see Methods).

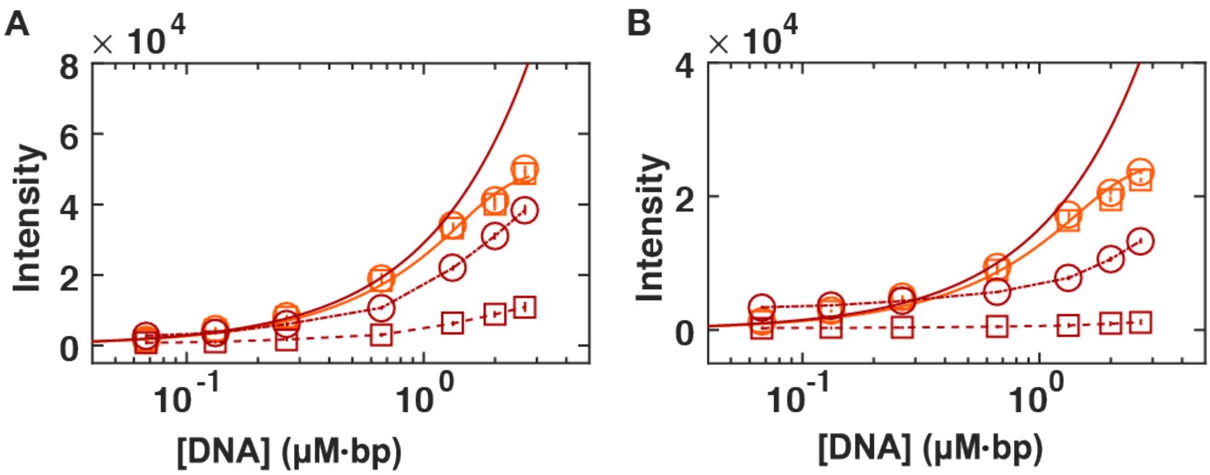


**Supplementary Figure S8. SYBR Gold fluorescence at high dye concentrations. A)** Fluorescence intensity recorded using a plate reader for torsionally unstrained DNA (pBR322). The orange circles and line are for [SYBR Gold] = 2.5 µM and identical to the data shown in Figure 4C, included as a reference. Red squares are raw data for [SYBR Gold] = 124 µM. Red circles are the same data corrected for the inner filter effect using Equation 10 and the path-lengths indicated in Figure 5. Dashed and dot-dashed lines are included as guides to the eye. The solid red line is the prediction for [SYBR Gold] = 124 µM using the finite concentration McGhee-von Hippel model and the parameters from Figure 4C. **B)** Fluorescence intensities recorded using a qPCR cycler. Same symbols, conditions, and fitting procedure as for the plate reader data shown in panel A.

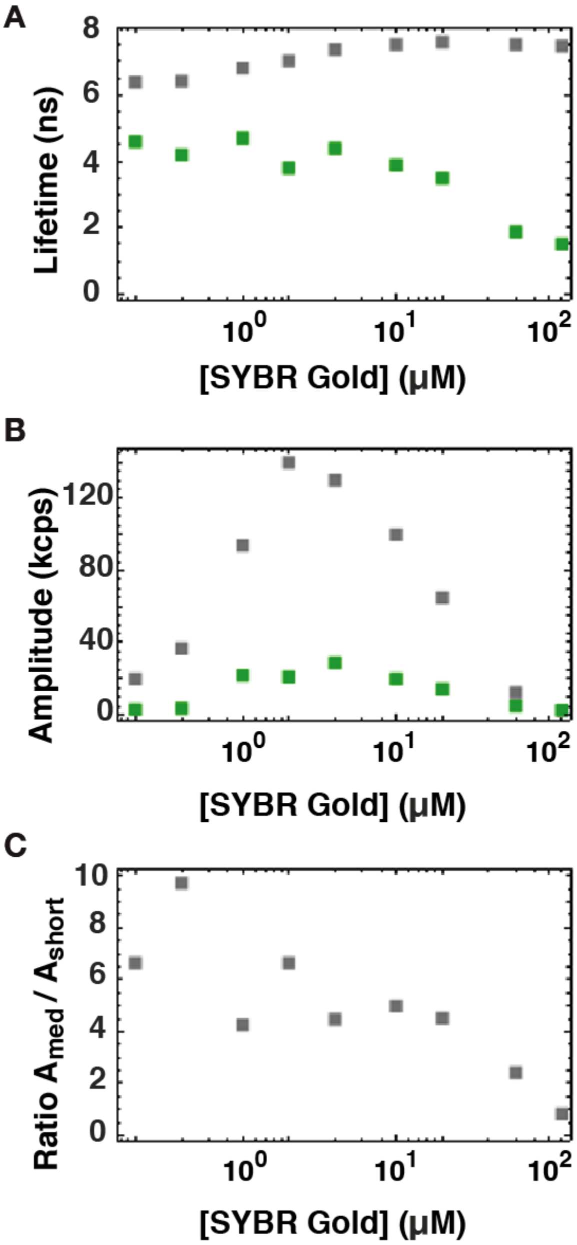


**Supplementary Figure S9. Fluorescence lifetime measurements. A)** Fluorescent lifetimes as a function of SYBR Gold concentration at a constant concentration of 2 µM·bp DNA determined from bi-exponential fits. These are the same data as shown in Figure 6B and D. The fit gives a shorter lifetime (green, ~2-4 ns) that decreases strongly with increasing SYBR Gold concentration and a longer lifetime (gray, ~6-7 ns) that slightly increases with increasing SYBR Gold concentration. **B)** Amplitudes of the two fluorescence lifetimes vs. SYBR Gold concentration; longer lifetime in green, shorter lifetime in grey. **C)** Ratio of the two amplitudes of the fluorescence lifetimes vs. SYBR Gold concentration. Up to concentrations of ~10 µM SYBR Gold, the long lifetime dominates the fit, with the short lifetime contributing < 25% of the total amplitude. At higher concentration, both amplitudes decrease as the total intensity strongly decreases, but the shorter lifetime –which we attribute to dynamic quenching from SYBR Gold molecules close to the DNA– becomes more important and roughly equal in magnitude to the long component.

## SUPPLEMENTARY REFERENCES


1. Evenson, W.E., Boden, L.M., Muzikar, K.A. and O'Leary, D.J. (2012) 1H and 13C NMR Assignments for the Cyanine Dyes SYBR Safe and Thiazole Orange. *The Journal of Organic Chemistry*, **77**, 10967-10971.
2. Wang, Y., Schellenberg, H., Walhorn, V., Toensing, K. and Anselmetti, D. (2017) Binding mechanism of PicoGreen to DNA characterized by magnetic tweezers and fluorescence spectroscopy. *European Biophysics Journal*, **46**, 561-566.
